# Near‐patient coagulation testing to predict bleeding after cardiac surgery: a cohort study

**DOI:** 10.1002/rth2.12024

**Published:** 2017-07-25

**Authors:** Andrew D. Mumford, Jessica Harris, Zoe Plummer, Kurtis Lee, Veerle Verheyden, Barnaby C. Reeves, Chris A. Rogers, Gianni D. Angelini, Gavin J. Murphy

**Affiliations:** ^1^ School of Cellular and Molecular Medicine University of Bristol Bristol UK; ^2^ Clinical Trials and Evaluation Unit University of Bristol Bristol UK; ^3^ University Hospitals Bristol NHS Foundation Trust, Bristol Bristol UK; ^4^ School of Clinical Sciences University of Bristol Bristol UK; ^5^ Department of Clinical Sciences University of Leicester Leicester UK

**Keywords:** blood coagulation, blood component transfusion, cardiac surgery, hemorrhage, point‐of‐care systems

## Abstract

**Essentials**

- Near‐patient testing improves coagulopathy diagnosis in cardiac surgery patients with severe bleeding.
- We investigated how well pre‐emptive near‐patient testing predicted severe bleeding.
- Severe bleeding could be predicted using both near‐patient tests and patient clinical characteristics.
- Near‐patient test results gave little additional predictive value over clinical characteristics alone.

**Background:**

Coagulopathic bleeding is common after cardiac surgery and is associated with increased morbidity, mortality and healthcare costs. Implementation of blood management algorithms in which patients with severe bleeding undergo near‐patient coagulation testing results in less overall bleeding and transfusion. However, it is unknown whether there is additional value from pre‐emptive near‐patient testing to predict whether severe bleeding will occur.

**Objectives:**

To evaluate how well a comprehensive panel of 28 near‐patient platelet and viscoelastometry tests predict bleeding after cardiac surgery, compared to prediction using baseline clinical characteristics alone.

**Methods:**

Single‐center, prospective cohort study in adults undergoing a range of cardiac surgery procedures. The primary outcome was clinical concern about bleeding (CCB), a composite of high blood loss (chest drain volume >600 mL within 6 hours), re‐operation for bleeding or administration of a pro‐haemostatic treatment directed by clinician judgement.

**Results:**

In 1833 patients recruited between March 2010 and August 2012, the median number of abnormal near‐patient test results was 5/28 per patient (range 0‐18). CCB occurred in 449/1833 patients (24.5%). The c‐statistic for a predictive model for CCB using only baseline clinical characteristics (*baseline‐only* model) was 0.72 (95% CI 0.69‐0.75). Addition of near‐patient test results to this model (*baseline‐plus‐test* model) improved the prediction of CCB (c‐statistic 0.75 [0.72‐0.77]), but increased the number of correctly classified patients by only 18 (0.98%).

**Conclusions:**

Near‐patient coagulation testing predicts bleeding in cardiac surgery patients, but offers little improvement in prediction compared to baseline clinical characteristics alone. trial registration: ISRNCTN 20778544 (http://www.isrctn.com/).

## 1 INTRODUCTION

Severe bleeding caused by coagulopathy is common after cardiac surgery and frequently requires large volume red blood cell (RBC) transfusion (>4 units) or emergency re‐operation.^1^, ^2^ Severe bleeding, RBC transfusion and re‐operation are independently associated with organ failure, sepsis and death.^3^, ^4^, ^5^ The provision of RBC and other blood components for cardiac surgery patients also has significant health care costs, and accounts for 10‐15% of the UK blood supply.^6^, ^7^

Near‐patient coagulation testing using viscoelastometry or rapid platelet function analysers detects the common sub‐types of coagulopathy associated with cardiac surgery in a clinically useful timescale.^8^, ^9^ Several small, single‐center, randomized controlled trials (RCTs) in cardiac surgery have shown that when compared to conventional laboratory testing or clinician judgement alone, near‐patient testing reduced transfusion of RBC^10^, ^11^ or non‐RBC blood components.^10^, ^11^, ^12^, ^13^, ^14^, ^15^ In a multicenter RCT of more than 7400 patients, implementation of a blood management algorithm incorporating near‐patient tests, resulted in reduced RBC transfusion, platelet transfusion and overall bleeding.^16^ In this trial, near‐patient test results were used to direct targeted treatments for coagulopathy only in patients who had already developed severe bleeding.^16^

Previous studies have also shown that some near‐patient test results from before the start, or immediately after the end of cardiac surgery also enable prediction of postoperative bleeding.^17^, ^18^, ^19^, ^20^, ^21^, ^22^, ^23^ This suggests an alternative blood management strategy in which near‐patient testing is performed pre‐emptively before the highest risk period for severe bleeding in the immediate post‐operative period. This is an attractive clinical strategy since identification of patients at the highest risk of bleeding could potentially enable selective targeted treatments to prevent severe bleeding starting. Predictive near‐patient testing has been incorporated into several blood management algorithms evaluated in several previous RCTs, usually as a single step for selection of preventative treatments^10^, ^12^, ^13^, ^15^ or in combination with later diagnostic near‐patient testing in patients who develop bleeding despite preventative treatments.^11^

Blood management algorithms incorporating near‐patient tests are recommended in US and European practice guidelines^24^, ^25^, ^26^, and are used widely.^27^ However, there is poor consensus about the best algorithm design, particularly whether near‐patient tests should be performed in response to severe bleeding, or whether there is additional value in pre‐emptive testing to help prevention of bleeding. We have performed a large prospective observational cohort study (Coagulation and Platelet Laboratory Testing in Cardiac Surgery [COPTIC] study; ISRCTN 20778544) to evaluate coagulation testing in a range of cardiac surgery procedures. In order to clarify the role of near‐patient testing, we now report an analysis of how well near‐patient tests predict bleeding, compared with prediction using patient clinical and procedural characteristics alone.

## 2 METHODS

### 2.1 Study design and patients

The COPTIC study was a single center, observational cohort study in which patients undergoing cardiac surgery at the Bristol Heart Institute were recruited in accordance with a pre‐specified protocol and in accordance with a UK NHS Research Ethics Committee approval (09/H0104/53). All patients aged over 18 years undergoing any non‐emergency cardiac surgical procedure were eligible unless they were prisoners or were unable to consent due to mental incapacity.

### 2.2 Care of patients and classification of pro‐haemostatic treatments

All participating patients gave written consent before surgery and were managed using standard anesthetic and surgical care pathways. Protamine (1 mg per 100 units of heparin) was given to reverse heparin anticoagulation immediately at the end of surgery. For procedures other than off‐pump coronary artery bypass grafting (CABG), patients received anti‐fibrinolytic drugs and additional protamine after the return of heparinized blood from the cardiopulmonary bypass circuit. These pro‐haemostatic treatments were classified as *directed by standard care* because the decision to treat was made before the start of surgery. Some patients also received pro‐haemostatic treatment with fresh frozen plasma, cryoprecipitate, platelets, recombinant Factor VIIa, fibrinogen concentrate, and additional anti‐fibrinolytic drugs or protamine because severe bleeding was judged to have started. These treatments were classified as *pro‐haemostatic treatments by clinical judgement*.

### 2.3 Baseline characteristic and near‐patient testing predictors

Patient clinical characteristics, surgical procedure and the results of conventional laboratory tests from pre‐operative assessments were recorded from electronic hospital records. Near‐patient tests were performed on a ‘pre‐operative sample’ obtained at induction of anesthesia and on a ‘post‐operative sample’ obtained at the end of surgery, after protamine for heparin reversal but before chest closure and insertion of chest drains.

All samples were tested using a multiple electrode aggregometry platelet function analyser (Multiplate; Roche Diagnostics, Rotkreuz, Switzerland, TRAP‐test, ASPI‐test,^,^ and ADP‐test reagents) and with adrenaline (ADR 100 mg mL^−1^). The post‐operative samples were also tested using a ROTEM delta thromboelastometer (TEM International GmbH, Munich, Germany; EXTEM, INTEM, HEPTEM, and FIBTEM reagents) and a TEG 5000 Thromboelastograph (Haemonetics Corp., Braintree, MA, USA; kaolin [CK] and kaolin/heparinise [CKH] reagents). Test results were unavailable to the clinicians responsible for the care of the patients.

### 2.4 Outcomes

The primary outcome was clinical concern about bleeding (CCB) after cardiac surgery, defined as a composite of any of the following: (i) a chest drain volume greater than 600 mL at 6 hours after admission to the cardiac intensive care unit (CICU); (ii) any re‐operation for bleeding during the hospital stay in which a surgical cause of bleeding was not identified; or, (iii) any *pro‐haemostatic treatment by clinical judgement* from the time of the post‐operative blood sample until 12 hours after CICU admission. Pro‐haemostatic treatments directed by clinical judgement were included in the primary outcome because these are the only reliable indicator of severe bleeding that occurs: (i) after the end of surgery but before chest drain insertion, or, (ii) after chest drain insertion but which is successfully reversed before the 600 mL chest drain volume threshold is reached.

The secondary outcomes were RBC transfusion, myocardial infarction (MI), stroke, acute kidney injury (AKI), sepsis and mortality (Table S1). The study observation period was the duration of hospital admission.

### 2.5 Selection of predictors

The baseline characteristics age, sex, diabetes, type of procedure, anti‐platelet drugs, surgical priority, estimated glomerular filtration rate, haematocrit, platelet count, and body mass index were selected as candidate predictors of CCB, before generation of predictive models (Table S2). Since a large number of different surgical procedures were performed in the study population, patients were classified into 12 categories depending on the type of procedure (CABG, CABG+valve, valve only, other high risk bleeding procedure) and pre‐operative anti‐platelet drugs (no anti‐platelet drugs, aspirin alone and aspirin+P2Y_12_ blocker sub‐grouped according to duration of omission of P2Y_12_ blocker before surgery: Table S2).

The candidate near‐patient test predictors were a panel of 28 results from pre‐operative or post‐operative MEA platelet function analysis or from post‐operative ROTEM or TEG viscoelastometry (Table S3). The viscoelastometry tests included measures of the speed of clot formation (INTEM/EXTEM clot time and α angle; CK R time), clot strength (INTEM/EXTEM maximum clot firmness; CK maximum amplitude), fibrinogen component of clot strength (FIBTEM maximum clot firmness), heparin effect (INTEM clot time ‐ HEPTEM clot time and CK R time ‐ CKH R time) and fibrinolysis (INTEM/EXTEM minimum lysis; CK lysis 60), which are previously reported coagulopathies after cardiac surgery.^8^, ^9^ The MEA platelet function analyzer ADP‐test and ASPI‐test results measures platelet dysfunction associated with P2Y_12_ blockers and aspirin, respectively.^28^ The EXTEM maximum clot firmness ‐ FIBTEM maximum clot firmness and TRAP‐test results were selected to measure global platelet dysfunction function.^11^, ^16^ Test results were incorporated into predictive models for CCB as continuous variable. However, in order help describe the distributions for the test results, each result was also classified as above or below a 95% reference interval obtained by from 42 healthy volunteers (median age 48 years, 68% male), determined locally using the same analyzers as the main study.

### 2.6 Statistical analysis

In order to compare predictive models that incorporated near‐patient test results with alternative models that included the baseline characteristics, the analysis population was defined as all patients with complete data for all predictors. Bias due to this constraint was investigated by calculating standardized mean differences (SMD)^29^ to compare the analysis population with those excluded because of missing data.

Logistic regression was used to develop predictive models and to estimate associations between CCB or secondary outcomes and the baseline characteristics (*baseline‐only* model) or alternative models that also included near‐patient test results (*baseline‐plus‐test* models). The near‐patient tests were further evaluated post hoc, with a model that included only the best‐fitting near‐patient test results (*test‐only* model). The best *baseline‐only, baseline‐plus‐test,* and *test‐only* models were selected as the models with the highest c‐statistic. Predictive value was also expressed as the proportion of patients correctly classified as CCB or no CCB, where those with a predicted probability of CCB ≥0.5 were classified as CCB. For all models, multivariable fractional polynomial techniques were used to investigate the linearity of terms. Model fit was assessed with Hosmer‐Lemeshow goodness‐of‐fit tests and individual contributions to the models were evaluated using likelihood ratio tests.

For the best *baseline‐plus‐test* model, three sensitivity analyses considered alternative formulations of the primary outcome (Table S4). In the first two sensitivity analyses, patients who were classified as CCB solely because of a *pro‐haemostatic treatment by clinical judgement* were either (i) excluded from analysis (SA1); or (ii) reclassified as no CCB (SA2). In a third post hoc sensitivity analysis, patients classified as CCB solely because they were transfused with 1‐2 units of plasma or with 1 unit of platelets, were reclassified as no CCB (SA3).

The c‐statistics from the primary outcome models were internally validated by bootstrapping with 1000 replicates and cross‐validated by removing one observation and then using the remaining analysis population to create models that generated the predicted probability of CCB for that one observation. After repeating the process for each observation, the predicted probabilities were used to build receiver operator characteristic curves and to calculate c‐statistics. All analyses were performed in STATA (version 14.0; STATA Corp, College Station, TX, USA).

## 3 RESULTS

### 3.1 Study population and primary outcome

**Figure 1:**
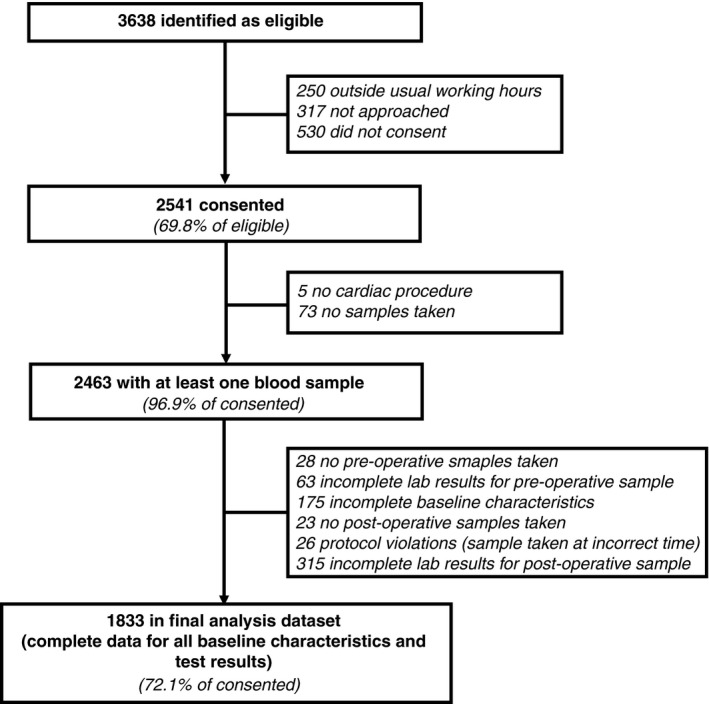
Study flow diagram

A total of 3638 patients were identified as eligible between March 2010 and August 2012, of which 2541 (69.8%) provided consent to participate (Fig. 1). The analysis population comprised 1833 patients (72.1% of consented) with complete baseline characteristics and near‐patient test results. This population had median age 68.9 years (range 18.7‐91.96) and included 1389 males (75.8%). Most of the analysis population underwent CABG with aspirin (40.2%), valve replacement with no anti‐platelet drugs (16.4%), or valve replacement with aspirin (7.3%; Table 1). Compared to the analysis population, the 708 patients excluded due to missing data had a smaller proportion of patients who underwent CABG with aspirin (31.7% vs 40.2%; SMD 0.18) and a higher proportion who underwent valve replacement with aspirin (10.3% vs 7.3%; SMD 0.11). For all the other baseline characteristics, the SMDs were less than 0.10, indicating that the groups were similar (Table S5).^30^

**Table 1:** Baseline characteristics of the analysis population (n = 1833)

Age (median, range)	68.9 (18.7, 91.9)
Sex; n (%) male	1389 (75.8%)
Diabetes; n (%) yes	380 (20.7%)
Procedure and anti‐platelet drugs; n (%)	
CABG: ASP	737 (40.2%)
CABG: no APT	61 (3.3%)
CABG+valve: no APT	66 (3.6%)
Valve: no APT	301 (16.4%)
CABG+valve: ASP	127 (6.9%)
Valve: ASP	133 (7.3%)
CABG: DAPT (0‐2 days)	98 (5.3%)
CABG: DAPT (3‐5 days)	114 (6.2%)
CABG: DAPT (6‐7 days)	88 (4.8%)
CABG+valve: DAPT (≤7 days)	14 (0.8%)
Valve: DAPT (≤7 days)	5 (0.3%)
Other high risk procedure	89 (4.9%)
Operative priority; n (%)	
Elective	1204 (65.7%)
Urgent	629 (34.3%)
eGFR (mL/min/1.73 m^2^; median, range)	73.7 (8.0, 214.6)
Pre‐operative haematocrit (%; median, range)	36.0 (19.0, 53.00)
Pre‐operative platelet count (×10^9^/L; median, range)	205.0 (43.0, 561.0)
Body mass index (kg/m^2^; mean, SD)	28.0 (4.7)

CABG, coronary artery bypass grafting; Valve, valve replacement; ASP, aspirin or aspirin plus P2Y_12_ blocker stopped more than 7 days before surgery; APT, any anti‐platelet drugs; DAPT, aspirin plus P2Y_12_ blocker 7 or less days before surgery shown with duration of omission of P2Y_12_ blocker before surgery shown in brackets; eGFP, estimated glomerular filtration rate; SD, standard deviation.

The primary outcome of CCB occurred in 449 (24.5%) of the analysis population, with 182 (9.7%) patients having more than one qualifying component. Considering the components separately, 362 (80.6%) of patients with CCB received a pro‐haemostatic treatment by clinical judgement, 244 (54.3%) had a chest drain volume greater than 600 mL and 57 (12.7%) had re‐operation for bleeding (Fig. S1).

### 3.2 Near‐patient coagulation test results

When compared to a 95% reference interval (RI) from a group of healthy controls not receiving anti‐platelet drugs, the analysis population had an overall median of 5 abnormal test results per patient (range 0/28‐18/28). The most commonly abnormal test results were reduced platelet function with the ASPI‐test (76.1% pre‐operative results below RI; 92.1% post‐operative results below RI), adrenaline (84.4% pre‐operative results below RI; 80.3% post‐operative below RI), and with the ADP‐test (24.5% pre‐operative results below RI; 55.1% post‐operative results below RI; Table S6). Abnormal viscoelastometry tests were less common (median 0 abnormal results per patient; range 0/28‐12/28), but included reduced ROTEM INTEM α‐angle (15.3% post‐operative results below RI) and FIBTEM maximum clot firmness (12.1% post‐operative results below RI) and reduced TEG maximum amplitude (12.4% postoperative results below RI; Table S6). The distributions of the near‐patient test results in the patients with, and without the primary outcome of CCB are shown in Fig. S2.

### 3.3 Prediction of CCB using baseline characteristics

**Figure 2:**
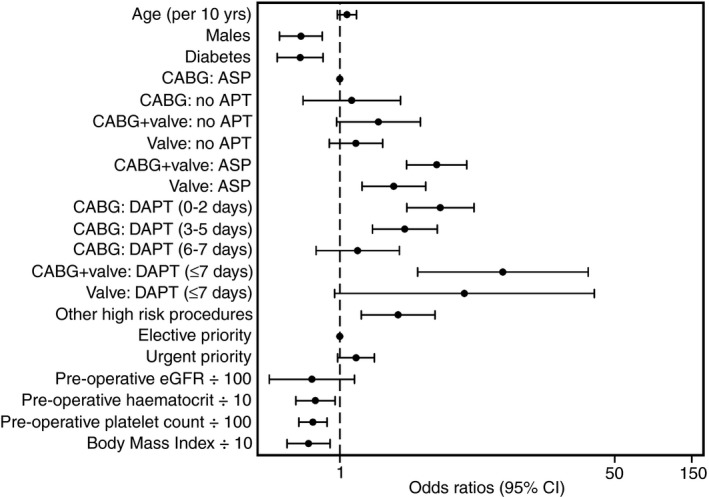
Baseline characteristics and clinical concern about bleeding. Data are odds ratios with 95% confidence intervals for clinical concern about bleeding. Odds ratios are for male vs female, presence of diabetes vs no diabetes, procedure type/anti‐platelet drug group vs CABG with ASP, and urgent vs elective priority. Odds ratios are adjusted for other factors in the table and for whether the patient was in an interventional study at our center. CABG, coronary artery bypass grafting; Valve, valve replacement; ASP, aspirin alone or aspirin plus P2Y_12_ blocker stopped for longer than 7 days before surgery; APT, any anti‐platelet drugs; DAPT, aspirin plus recent P2Y_12_ blocker stopped 7 days or less before surgery, with duration of omission of P2Y_12_ blocker indicated in brackets. Odds ratios and 95% confidence intervals are also reported in Table S7

**Figure 3:**
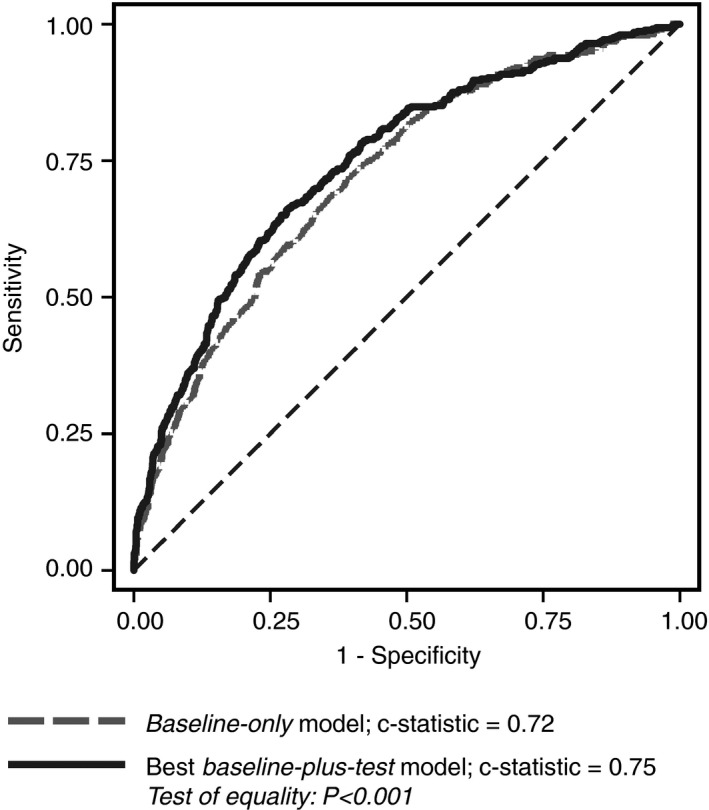
Receiver operator characteristics curve for the *baseline‐only* and best *baseline‐plus‐test* models for prediction of clinical concern about bleeding. This *baseline‐plus‐test* model incorporated near‐patient test result from post‐operative TEG and post‐operative MEA analyzers

Baseline characteristics relating to sex, diabetes, procedure/anti‐platelet drugs, haematocrit, platelet count, and body mass index were statistically significant independent predictors of CCB. Most of the variation in CCB was accounted for by procedure/anti‐platelet drugs. CABG with aspirin was the largest group and was the reference category. Compared with this group, CABG+valve and valve procedures in patients receiving dual anti‐platelet treatment (aspirin plus P2Y_12_ blocker within 7 days or less of surgery) conferred the highest odds of CCB (Fig. 2, Table 2). The predictive model for CCB using the baseline characteristics (*baseline‐only* model), had a c‐statistic of 0.72 (0.69‐0.75; Fig. 3, Table S7) and correctly classified 76.8% of patients as CCB or no CCB. The results of the internal validation and goodness of model fit are shown in Table S7.

**Table 2:** Baseline characteristics and CCB

	No CCB; n=1384	CCB; n=449	Adjusted OR^a^	*P*‐value
Age (median, range)	68.1 (18.7, 91.9)	71.3 (19.5, 91.4)	1.01 (1.00, 1.02)	.14
Sex; n (%) male	1029 (74.3%)	360 (80.2%)	0.57 (0.42, 0.78)	<.001
Diabetes; n (%)	314 (22.7%)	66 (14.7%)	0.57 (0.41, 0.79)	.001
Procedure and anti‐platelet medication; n (%)				
CABG: ASP	619 (44.7%)	118 (26.3%)	1.00	<.001
CABG: no APT	50 (3.6%)	11 (2.4%)	1.19 (0.59, 2.37)	
CABG+valve: no APT	47 (3.4%)	19 (4.2%)	1.73 (0.95, 3.15)	
Valve: no APT	239 (17.3%)	62 (13.8%)	1.26 (0.86, 1.84)	
CABG+valve: ASP	68 (4.9%)	59 (13.1%)	3.97 (2.59, 6.10)	
Valve: ASP	90 (6.5%)	43 (9.6%)	2.16 (1.37, 3.39)	
CABG: DAPT (0‐2 days)	58 (4.2%)	40 (8.9%)	4.18 (2.59, 6.75)	
CABG: DAPT (3‐5 days)	76 (5.5%)	38 (8.5%)	2.52 (1.59, 4.00)	
CABG: DAPT (6‐7 days)	72 (5.2%)	16 (3.6%)	1.29 (0.71, 2.33)	
CABG + valve: DAPT (≤7 days)	4 (0.3%)	10 (2.2%)	10.18 (3.02, 34.33)	
Valve: DAPT (≤7 days)	2 (0.1%)	3 (0.7%)	5.89 (0.93, 37.47)	
Other high risk procedure	59 (4.3%)	30 (6.7%)	2.29 (1.36, 3.88)	
Priority n (%)				
Elective	928 (67.1%)	276 (61.5%)	1.00	.09
Urgent	456 (32.9%)	173 (38.5%)	1.26 (0.97, 1.64)	
Pre‐operative eGFR (mL/min/1.73 m^2^; median, range)	75.7 (8.0, 214.6)	68.4 (12.0, 196.6)	1.00 (0.99, 1.00)	.20
Pre‐operative haematocrit (%; mean, SD)	36.1 (4.2)	35.4 (4.8)	0.97 (0.94, 0.99)	.01
Pre‐operative platelet count (×10^9^/L; median, range)	207.5 (62.0, 561.0)	196.0 (43.0, 548.0)	1.00 (0.99, 1.00)^b^	<.001
Body mass index (kg/m^2^; median, range)	27.7 (16.5, 55.5)	26.6 (16.2, 64.5)	0.96 (0.93, 0.99)	.004

CABG, coronary artery bypass grafting; Valve, valve replacement; CABG+valve, combined CABG and valve replacement; ASP, pre‐operative aspirin; no APT, no pre‐operative anti‐platelet medication; DAPT, aspirin+P2Y_12_ blocker shown with duration of omission of P2Y_12_ blocker before surgery; eGFR, estimated glomerular filtration rate.

a Odds ratios are adjusted for all other factors in the table, and for whether the participant was included in an interventional study at our center. The *P* values were calculated from this multivariate analysis.

b The absolute OR for platelet count expressed as ×109/L is 0.9961738 (95% CI 0.9941638, 0.9981878).

### 3.4 Prediction of CCB using baseline characteristics and near‐patient test results

Alternative predictive models incorporating near‐patient test results altered the prediction of CCB compared to the baseline‐only model (Table S8). The best *baseline‐plus‐test* model incorporated baseline characteristics plus the post‐operative MEA platelet function results with the ASP‐test and adrenaline and the TEG maximum amplitude (Table S9), and had a c‐statistic of 0.75 (0.72‐0.77; *P*=.001 compared to *baseline‐only* model; Fig. 3, Table S8). Application of the best *baseline‐plus‐test* model resulted in correct reclassification of 63 patients (49 who had CCB who were initially classified as no CCB and 14 who did not have CCB but were initially classified as no CCB). However, 45 patients were incorrectly reclassified (6 who had CCB who were initially classified as CCB and 39 who did not have CCB who were initially classified as no CCB). Therefore, the net improvement of correct classification was only 18/1833 patients (0.98%). The internal validation and goodness of model fit are shown in Table S7.

### 3.5 Sensitivity analyses

After excluding (SA1) or re‐classifying (SA2) 181 patients who were classified as having CCB solely because they had received a pro‐haemostatic treatment by clinician judgement (SA1: CCB in 268/1652 [16.2%]; SA2: CCB in 268/1833 [14.6%]), the c‐statistics of the best *baseline‐plus‐test* model did not differ from the original CCB definition (Table S8). In the *post hoc* sensitivity analysis (SA3), in which patients who received only small volume plasma or platelet transfusions were reclassified as no CCB (CCB in 348/1832 [19.0%]), there was also no difference in c‐statistic (Table S8).

### 3.6 Prediction of CCB using only near‐patient test results

**Figure 4:**
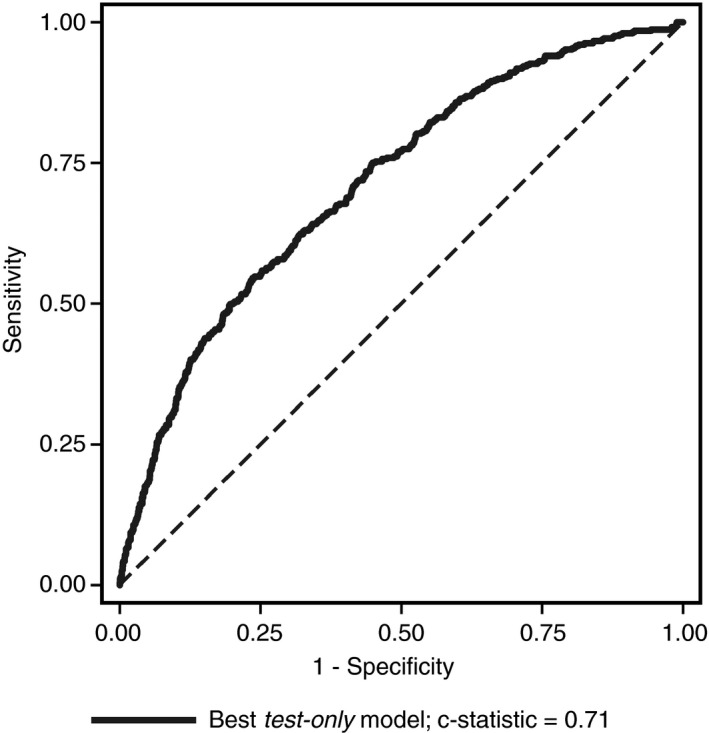
Receiver operator characteristics curve for the best *test‐only* model for prediction of clinical concern about bleeding. This model incorporated near patient test result from the pre‐operative MEA, post‐operative MEA and post‐operative TEG analysers

The best *test‐only* model for CCB, which included only the near‐patient test results without baseline characteristics, had a c‐statistic of 0.71 (0.69‐0.74; Fig. 4, Table S7).

### 3.7 Secondary outcomes

A total of 660/1797 (36.7%) of the analysis population received at least a one unit RBC transfusion, and 96/1796 (5.4%) received more than four units before CICU discharge. The prevalence of all the secondary outcomes are shown in Table 3 and their relationships with the near‐patient test results in Table S10. Inclusion of near‐patient test results into *baseline‐only* models generated for each secondary outcome marginally improved prediction for any post‐operative RBC transfusion (c‐statistic 0.85 [0.83‐0.87] for *baseline‐plus‐test* vs 0.83 (0.81‐0.85) for *baseline‐only*;* P*<.001) and for sepsis (c‐statistic 0.74 [0.70‐0.79] for *baseline‐plus‐test* vs 0.70 [0.65‐0.75] for *baseline‐only*;* P*=.01). Inclusion of near‐patient test results did not improve the c‐statistics for the other secondary outcomes (Table 3).

**Table 3:** Performance of predictive models for the secondary outcomes

Secondary outcome	Frequency	*Baseline‐only* model c‐statistic (95% CI)	B*aseline‐plus‐test* model c‐statistic (95% CI)	*P* value
RBC intra‐op/post‐op (0 vs ≥1 u)	660/1797 (36.7%)	0.85 (0.83‐0.87)	0.85 (0.83‐0.87)	.58
RBC intra‐op/post‐op (≤4 vs >4 u)	96/1796 (5.4%)	0.85 (0.81‐0.89)	No change	–
RBC post‐op (0 vs ≥1 u)	581/1795 (32.4%)	0.83 (0.81‐0.85)	0.85 (0.83‐0.87)	<.001
RBC post‐op (≤4 vs >4 u)	59/1802 (3.3%)	0.83 (0.78‐0.88)	0.84 (0.78‐0.89)	.44
Death	34/1833 (1.9%)	0.84 (0.77‐0.91)	No change	–
Myocardial infarction	19/1828 (1.0%)	0.68 (0.55‐0.82)	No change	–
Stroke	19/1828 (1.0%)	0.81 (0.72‐0.90)	No change	–
Acute kidney injury	819/1833 (44.7%)	0.76 (0.74‐0.78)	0.76 (0.74‐0.79)	.08
Sepsis	104/1826 (5.7%)	0.70 (0.65‐0.75)	0.74 (0.70‐0.79)	.01

RBC, red blood cell transfusion. C‐statistics are shown for the *baseline‐only* model that incorporates the demographic and clinical characteristics of the patients and the best *baseline‐plus‐test* model that also incorporates near‐patient laboratory test results. Only pre‐operative test results were considered for the intra‐op/post‐op outcomes.

## 4 DISCUSSION

In this large, prospective, observational cohort study we evaluated whether comprehensive near‐patient coagulation testing improved prediction of severe bleeding after cardiac surgery when compared with prediction based on patient baseline characteristics alone. The first main finding was that the baseline characteristics of patients enabled a clinically useful level of prediction of the primary outcome of CCB (*baseline‐only* model*)*. Abnormal near‐patient tests were common in the analysis population. However, including the test results into a predictive model that already contained baseline characteristics (*baseline‐plus‐test* model) resulted in the correct reclassification of only 0.98% of patients as either CCB or no CCB, suggesting minimal clinical benefit. Including near‐patient test results into *baseline‐only* models for the secondary outcomes of blood component use, post‐operative complications and mortality also gave little improvement in prediction.

### 4.1 Comparison with other studies

The *baseline‐only* model developed in the analysis population enabled the correct classification of 76.8% of patients as either CCB or no CCB. This is consistent with several previous studies in which demographic details, comorbidities, conventional pre‐operative laboratory test results^31^, ^32^, ^33^, ^34^, ^35^, and procedural characteristics^31^, ^35^ contributed to prediction of RBC transfusion. The *baseline‐only* model confirmed that exposure to anti‐platelet drugs before cardiac surgery is also a strong predictor of post‐operative bleeding.^17^, ^20^ We also showed that for patients having CABG, aspirin plus a P2Y_12_ blocker stopped for less than 2 days increased the risk of severe bleeding fourfold compared to aspirin alone. However, there was no increased risk if the P2Y_12_ blocker was stopped for 6 to seven days. This confirms the empiric advice in current practice guidelines^36^, that stopping P2Y_12_ blockers for at least 5 days achieves the greatest reduction in bleeding risk.

A unique feature of the COPTIC study was the comprehensive panel of near‐patient tests used to detect coagulopathy. One striking finding was that the analysis population had a median of 5/28 test results per patient that were abnormal (range 0‐18) when compared to healthy control reference intervals, which were similar in our study to previously published reference intervals.^37^, ^38^, ^39^By far the most common abnormalities were reduced platelet function with the ASPI‐test and adrenaline reagents. Since both reagents are sensitive to dysfunction of the platelet cyclooxygenase pathway^28^, ^40^, this finding is consistent with the high proportion of patients receiving aspirin (approximately 75%). Reduced platelet function with the ADP‐test was less common, reflecting the lower number of patients with any exposure to P2Y_12_ blockers before surgery (approximately 20%).^28^

Compared to the platelet function tests, abnormal viscoelastometry tests were less common (median 0/28 abnormal tests per patient; range 0‐12), but included reduced ROTEM INTEM α‐angle, ROTEM FIBTEM maximum clot firmness and reduced TEG maximum clot firmness. Together, these suggest impaired clot formation and fibrinogen component of clot strength, both previously reported after cardiac surgery.^41^, ^42^Near‐patient test results indicating reduced thrombin generation, heparin‐effect or hyperfibrinolysis were uncommon in the analysis population.

Although addition of near‐patient test results to the *baseline‐only* model gave a statistically significant increase in prediction, there was only a small benefit when expressed as the proportion of patients correctly classified as CCC or no CCB. In a post hoc analysis, a *test‐only* model also predicted CCB with a similar c‐statistic to the *baseline‐only* model. Therefore, the failure of near‐patient tests to substantially improve prediction does not indicate that testing per se does not predict bleeding. Instead, it is likely that most of the predictive information from near‐patient testing is already provided by the patients’ baseline characteristics. The abundance of abnormal platelet function test results in the analysis population suggests that this is particularly relevant for exposure anti‐platelet drugs.

### 4.2 Strengths and weaknesses

The main strength of the COPTIC study was the low risk of bias enabled by (i) enrolment of unselected patients having a range of cardiac surgery procedures, (ii) enrolment of nearly 70% of all eligible patientsand, and (iii) standardized testing of blood samples remote from surgery so that results could not influence interventions or outcomes. The COPTIC study is also by far the largest and most comprehensive analysis of the predictive value of near‐patient tests in cardiac surgery.

A further strength was our definition of CCB as a composite endpoint to reflect severe bleeding. Our definition included high blood loss into chest drains and reoperation for bleeding, similar to other cardiac surgery studies.^43^ However, we also included *pro‐haemostatic treatments by clinical judgement* administered in response to observed severe bleeding, to identify patients that would not be captured with the other endpoint definitions. It is a potential criticism that some *pro‐haemostatic treatments by clinicial judgement* may have been given before severe bleeding was observed, resulting in misclassification of patients as CCB. However, in sensitivity analyses using more conservative definitions of severe bleeding for the primary outcome, the main findings remained consistent.

It is also a potential weakness of the study that some participants had one or more items of missing data, usually because of quality control failure of one or more tests or because of insufficient evaluable blood samples. To address this limitation, we decided to carry out a ‘complete case analysis’ in preference to imputing missing data because the study had adequate power when analyzing only those participants with complete data. The standardized mean differences of the baseline characteristics of the excluded population were similar to the analysis population, indicating that it is unlikely that this exclusion strategy introduced bias.

### 4.3 Clinical impact of study findings

Current practice guidelines support incorporating near‐patient coagulation testing into blood management algorithms for cardiac surgery.^24^, ^25^, ^26^ However, these guidelines do not distinguish between the use of near‐patient tests to inform treatment selection after patients develop severe bleeding, and the use of pre‐emptive testing to predict bleeding and to direct preventative treatments.

The results of the COPTIC study provide general support for near‐patient testing to assist blood management in cardiac surgery because our findings confirm that currently available near‐patient analyzers distinguish different coagulopathies in this setting. Identifying high bleeding risk patients before bleeding occurs so that targeted preventative treatments can be given is an attractive potential clinical strategy. We have shown that bleeding can be predicted to a modest extent by considering clinical baseline characteristics that are readily available before the start of surgery, and that there is unlikely to be any additional benefit from pre‐emptive near‐patient testing in unselected patients.

## RELATIONSHIP DISCLOSURES

The study was funded by a Programme for Applied Health Research grant from the United Kingdom National Institute for Health Research (NIHR; RP/PG/0407/10384) and by the NIHR Bristol Biomedical Research Unit in Cardiovascular Disease. GJM is a British Heart Foundation (BHF) Professor of Cardiac Surgery and CAR is a BHF Professor in Medical Statistics and Clinical Trials. We wish to acknowledge the Bristol Clinical Trials and Evaluation Unit and the clinical cardiac surgery and anaesthetics staff at the Bristol Heart Institute for their contribution to the delivery of this study. The views and opinions expressed are those of the authors and do not necessarily reflect those of the NIHR, the BHF, the UK NHS, or the Department of Health.
