# Supplementary material for: Near‐patient coagulation testing to predict bleeding after cardiac surgery: a cohort study
Source: Res Pract Thromb Haemost. 2017 Jul 25;1(2):242–51. doi: 10.1002/rth2.12024 (PMC5992888; doi:10.1002/rth2.12024)

SUPPLEMENTARY FIGURES

Figure S1: Proportions of patients who reached the primary outcome of clinical concern about bleeding (n=449) subdivided according to the different components of the endpoint definition.

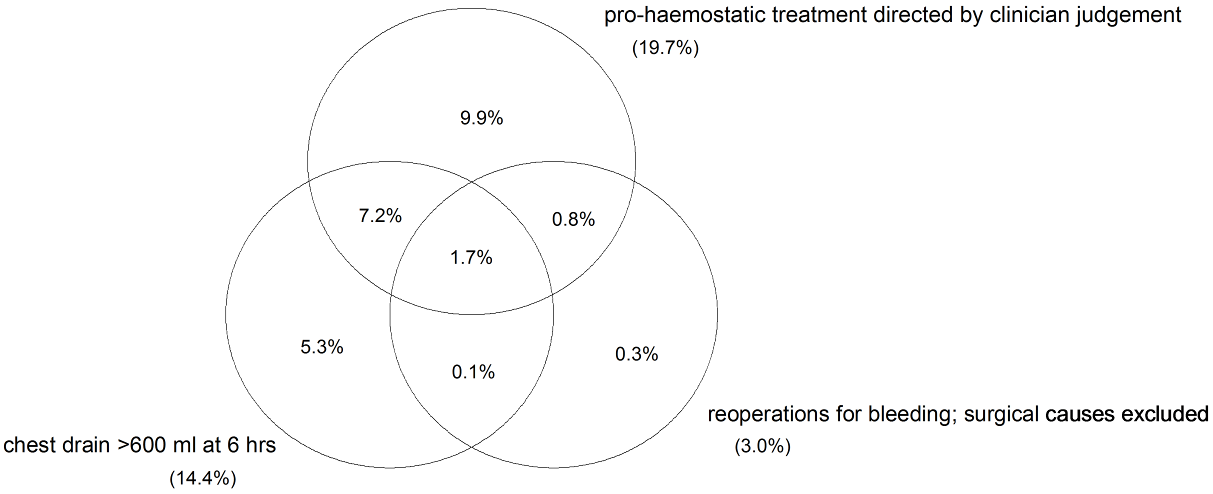

Figure S2: Distributions of near patient test results in the entire analysis population (n=1,833). Patients who reached the primary outcome of clinical concern about bleeding are indicated as black bars, and those who did not reach the primary outcome as white bars. AUC- area under curve; CT-clot time; α angle initial slope of clot formation; MCF- maximum clot firmness; ML- maximum clot lysis expressed as the percentage reduction of MCF onbserved at the end of the observation period of 60 minutes; V_{max} - maximum rate of increase in clot firmness; tV_{max}- time to reach maximum rate of increase in clot firmness; CK- TEG citrated kaolin test; CKH- TEG citrated kaolin with heparinase test; R- clot response time; MA clot maximum amplitude; LY 60 clot lysis at 60 minutes expressed as a percentage change in area of the thromboelastogtraphy curve between the point of MA and the end of the 60 minute observation period.

1. Multiple electrode platelet function analyser

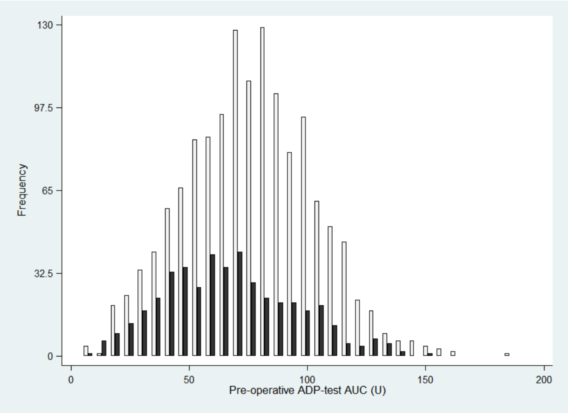

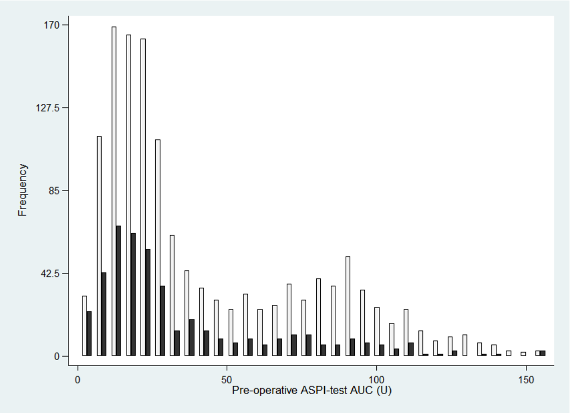

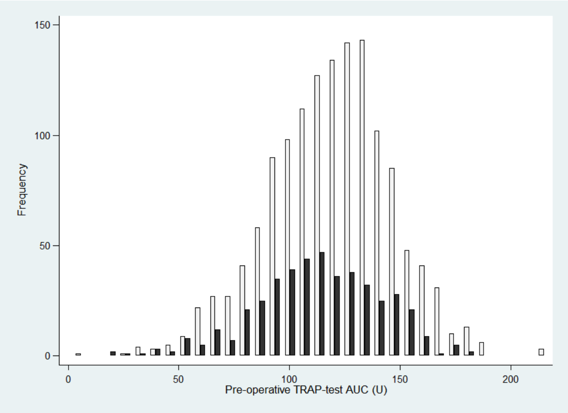

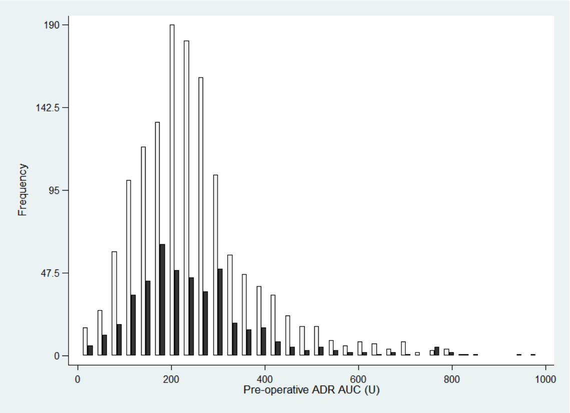

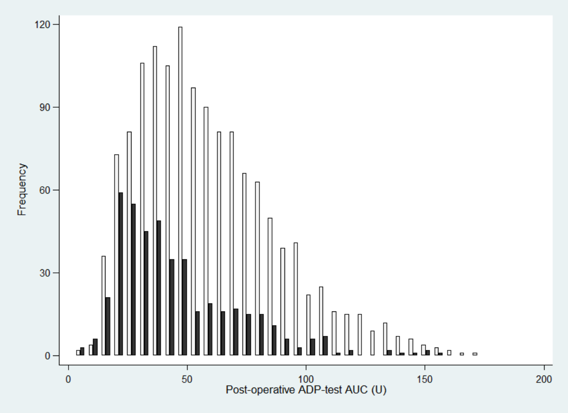

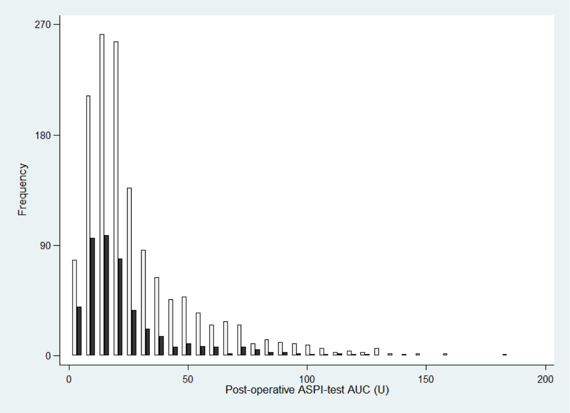

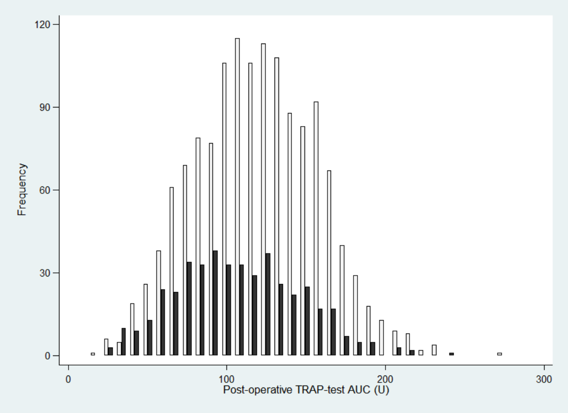

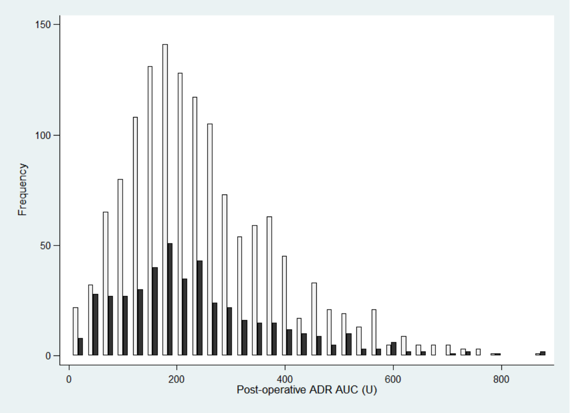

2. ROTEM delta thromboelastometer

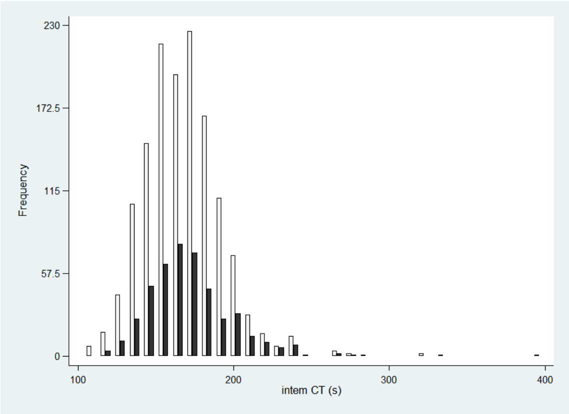

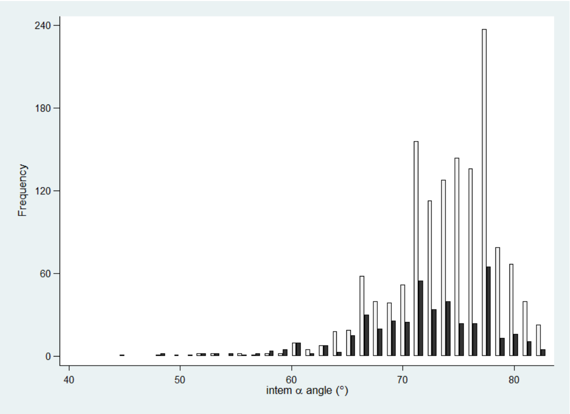

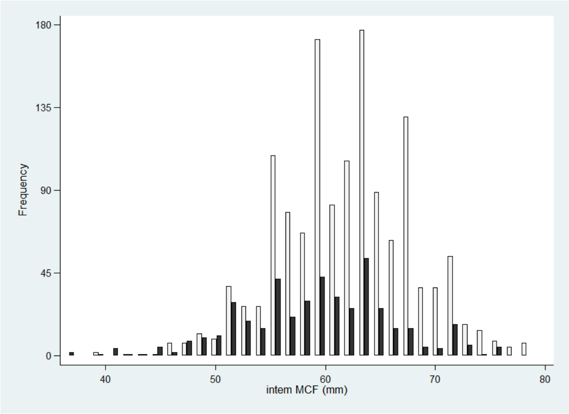

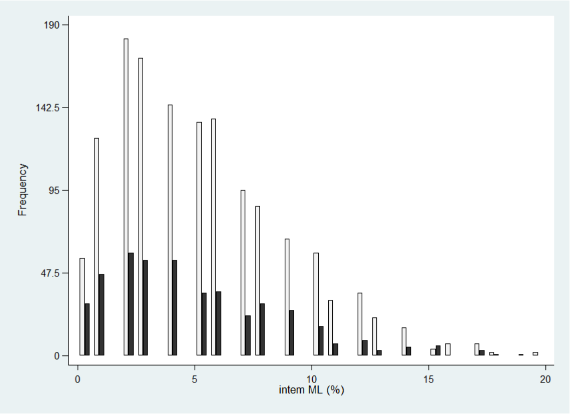

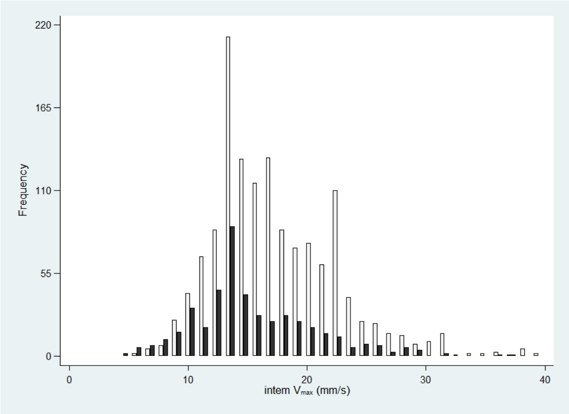

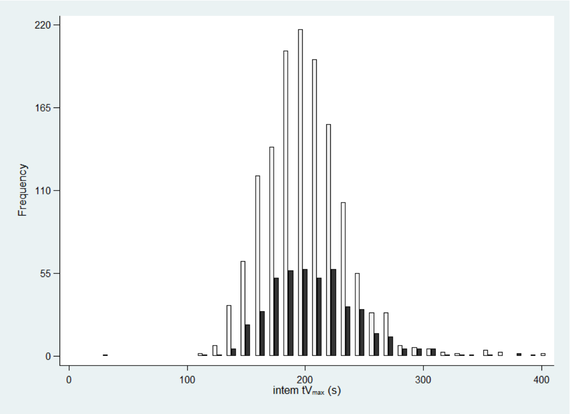

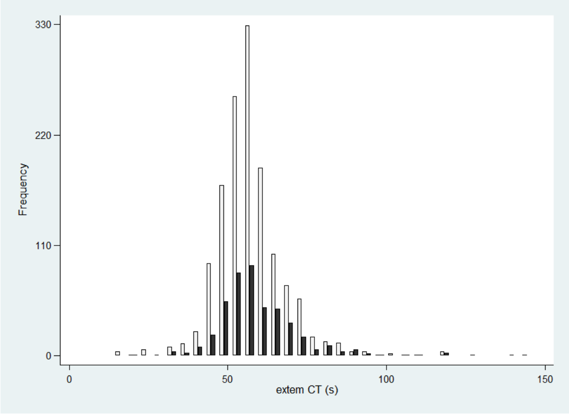

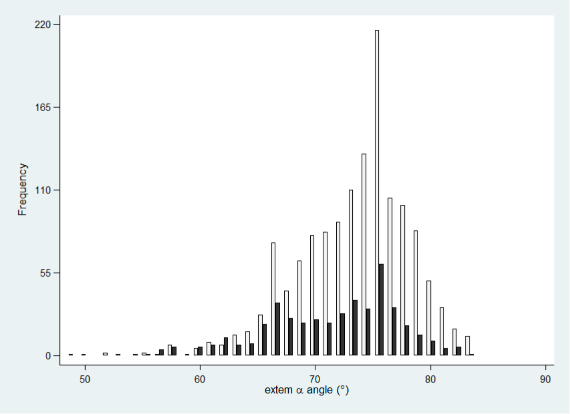

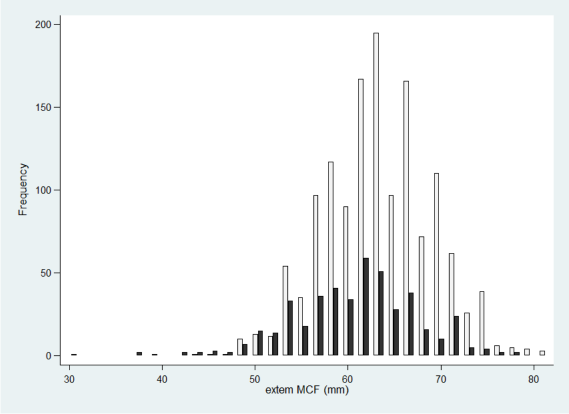
Frequency
0
50
100
150
200
30
40
50
60
70
80
extem MCF (mm)

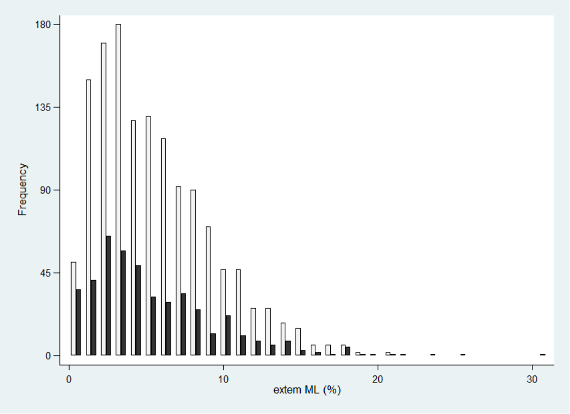
Frequency
0
45
90
135
180
0
10
20
30
extem ML (%)

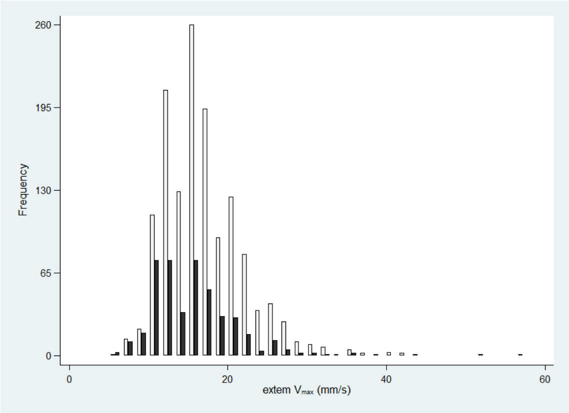
Frequency
0
65
130
195
260
0
20
40
60
extem V_{max} (mm/s)

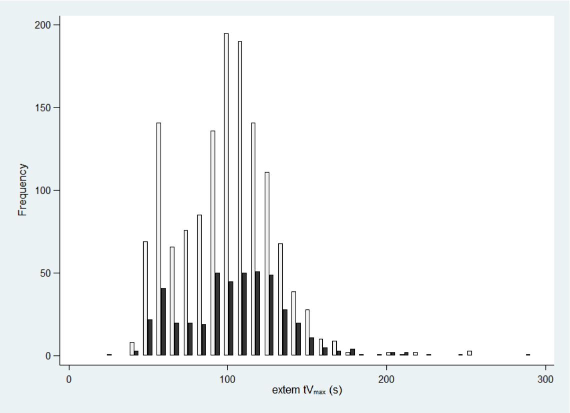
Frequency
0
50
100
150
200
0
100
200
300
extem tV_{max} (s)

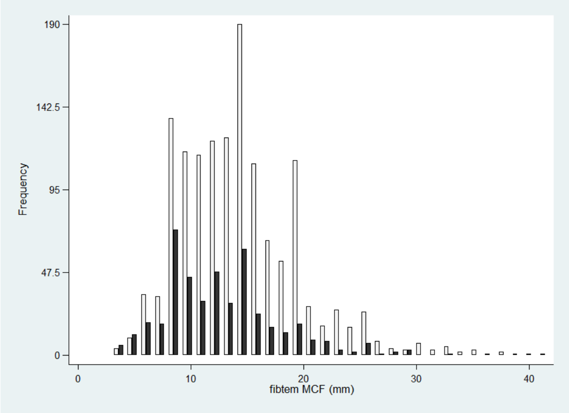
Frequency
0
47.5
95
142.5
190
0
10
20
30
40
fibtem MCF (mm)

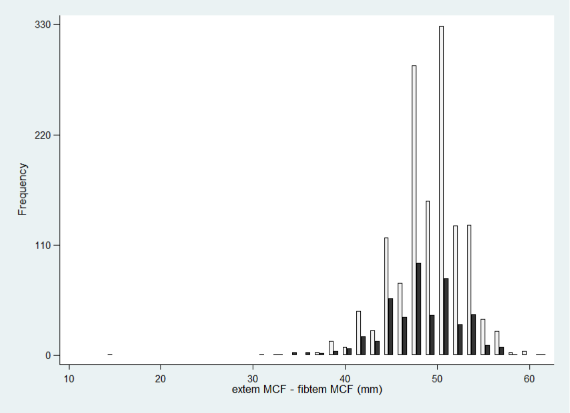
Frequency
0
110
220
330
10
20
30
40
50
60
extem MCF - fibtem MCF (mm)

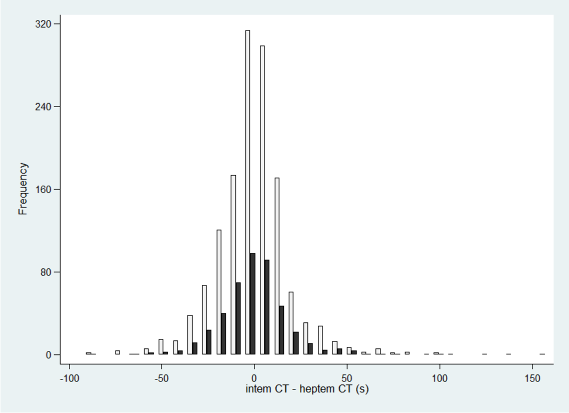
Frequency
0
80
160
240
320
-100
-50
0
50
100
150
intem CT - heptem CT (s)

3. TEG 5000 thromboelastograph

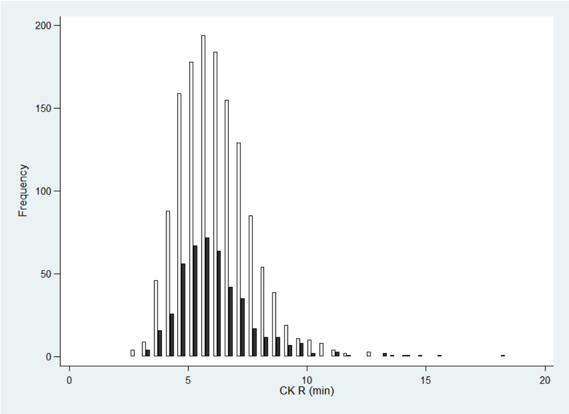

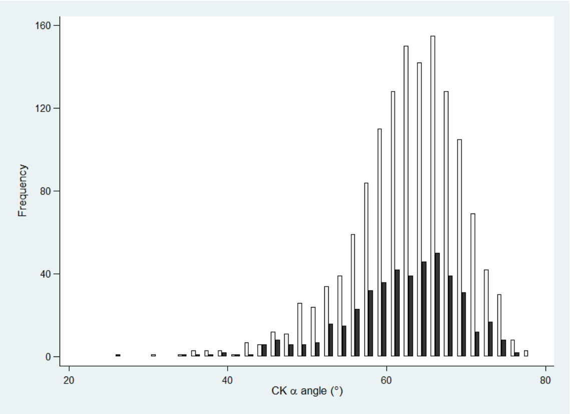

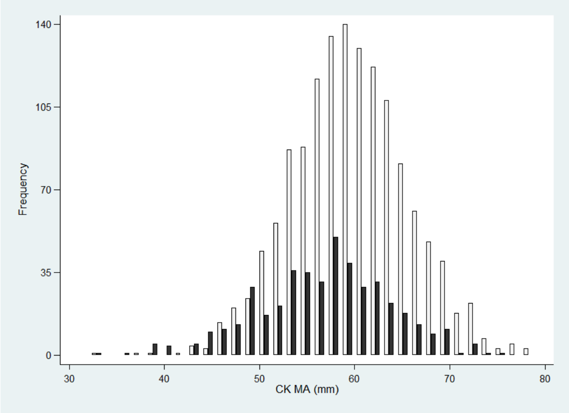

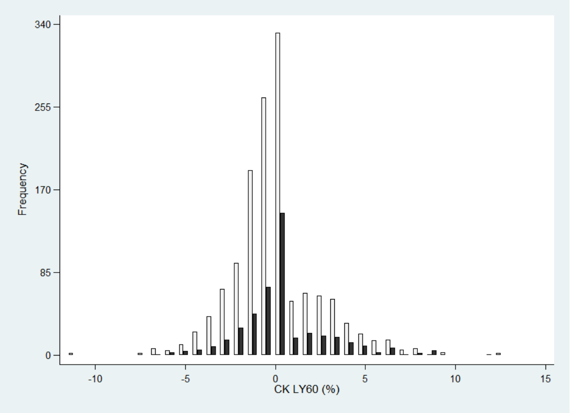

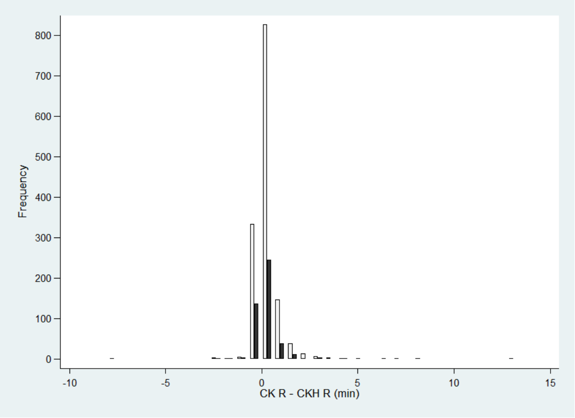

Supplement: Supplementary file 1 [file RTH2-1-242-s001.pdf]
