# Supplementary material for: Near‐patient coagulation testing to predict bleeding after cardiac surgery: a cohort study
Source: Res Pract Thromb Haemost. 2017 Jul 25;1(2):242–51. doi: 10.1002/rth2.12024 (PMC5992888; doi:10.1002/rth2.12024)
Supplement: Supplementary file 2 [file RTH2-1-242-s002.docx]

## Table S1: Secondary endpoint definitions.

| Red cell transfusion intra-op or post-op (0 vs ≥1 unit) | Any red cell transfusion between the time of the pre-op blood sample and discharge from CICU. |
| --- | --- |
| Red cell transfusion intra-operative or post-operaive (≤4 vs >4 units) | Any red cell transfusion >4 units between the time of the pre-op blood sample and discharge from CICU. |
| Red cell transfusion post-operative  (0 vs ≥1 unit) | Any red cell transfusion between the time of the post-op blood sample and discharge from CICU. |
| Red cell transfusion post-operative  (≤4 vs >4 units) | Any red cell transfusion >4 units between the time of the post-op blood sample and discharge from CICU. |
| Mortality | Death during hospital admission. |
| Myocardial infarction | Any ST elevation or non-ST elevation myocardial infarction occurring before discharge from hospital OR death from myocardial infarction in hospital. |
| Stroke | Any stroke, transient ischaemic attack, hemiplegia, paralysis, paraplegia, dysphasia, cerebral infarct or embolus occurring before discharge from hospital. |
| Acute kidney injury | Serum creatinine increased by ≥26.5 μmol/l from the pre-operative value within 48 hours of surgery OR serum creatinine increased ≥1.5 times the pre-operative value within 7 days of surgery. |
| Sepsis | Any chest infection; wound infection; mediastinitis; graft site infection; sternal wound infection; urinary tract infection; septic; sepsis; blood borne infection occurring before discharge from hospital. |

CICU- Cardiac intensive care unit
