# Supplementary material for: Near‐patient coagulation testing to predict bleeding after cardiac surgery: a cohort study
Source: Res Pract Thromb Haemost. 2017 Jul 25;1(2):242–51. doi: 10.1002/rth2.12024 (PMC5992888; doi:10.1002/rth2.12024)
Supplement: Supplementary file 3 [file RTH2-1-242-s003.docx]

**Table S2: Baseline characteristic predictors**

| **Age** (years) |
| --- |
| **Sex** (Male vs Female) |
| **Diabetes** (yes or no) |
| **Type of procedure/anti-platelet medication** |
| CABG: aspirin: no P2Y_12_ blocker or P2Y_12_ blocker stopped for >7 days  CABG: no anti-platelet medication |
|  |
| CABG + valve: no anti-platelet medication |
| Valve: no anti-platelet medication |
| CABG + valve: aspirin: no P2Y_12_ blocker or P2Y_12_ blocker stopped for >7 days |
| Valve: aspirin: no P2Y_12_ blocker or P2Y_12_ blocker stopped for >7 days |
| CABG: aspirin: P2Y_12_ blocker stopped for 0-2 days |
| CABG: aspirin: P2Y_12_ blocker stopped for 3-5 days |
| CABG: aspirin: P2Y_12_ blocker stopped for 6-7 days before |
| CABG + valve: aspirin: P2Y_12_ blocker stopped for ≤7 days |
| Valve only: aspirin: P2Y_12_ blocker stopped for ≤7 days |
| Other high bleeding risk procedure: any or no anti-platelet medication |
| **Surgical priority**  Elective  Urgent |
| **Pre-operative estimated glomerular filtration rate** (ml/min/1.73m^2^) |
| **Pre-operative haematocrit** (%) |
| **Pre-operative platelet count** (x10^9^/L) |
| **Body mass index** (kg/m^2^) |

The pre-operative characteristics relating to type of surgical procedure, type of anti-platelet medication and duration of withdrawal of P2Y_12_ blocker were grouped into 12 categories in order to reduce the number of degrees of freedom in the predictive models and to avoid groupings with zero participants. CABG- coronary artery bypass graft surgery; Valve- valve replacement surgery; CABG + valve- combined CABG and valve replacement; P2Y_12_ blocker- clopidogrel or prasugrel.
