# Supplementary material for: Near‐patient coagulation testing to predict bleeding after cardiac surgery: a cohort study
Source: Res Pract Thromb Haemost. 2017 Jul 25;1(2):242–51. doi: 10.1002/rth2.12024 (PMC5992888; doi:10.1002/rth2.12024)
Supplement: Supplementary file 4 [file RTH2-1-242-s004.docx]

**Table S3: Near patient test predictors.**

| **Test group** | **Test** | **Units** |
| --- | --- | --- |
| **Pre-operative MEA platelet function** | ADP-test AUC | Aggregation units (U) |
|  | ASPI-test AUC | Aggregation units (U) |
|  | TRAP-test AUC | Aggregation units (U) |
|  | ADR AUC | Aggregation units (U) |
| **Post-operative MEA platelet function** | ADP-test AUC | Aggregation units (U) |
|  | ASPI-test AUC | Aggregation units (U) |
|  | TRAP-test AUC | Aggregation units (U) |
|  | ADR AUC | Aggregation units (U) |
| **Post-operative ROTEM** | Intem CT | s |
|  | Intem α angle | degrees |
|  | Intem MCF | mm |
|  | Intem ML | % reduction in MCF |
|  | Intem V_max_ | mm/s |
|  | Intem tV_max_ | s |
|  | Extem CT | s |
|  | Extem α angle | degrees |
|  | Extem MCF | mm |
|  | Extem ML | % reduction in MCF at end of observation interval |
|  | Extem V_max_ | mm/s |
|  | Extem tV_max_ | s |
|  | Fibtem MCF | mm |
|  | Extem MCF - Fibtem MCF | mm |
|  | Intem CT - Heptem CT | s |
| **Post-operative TEG** | TEG CK R | min |
|  | TEG CK α angle | degrees |
|  | TEG CK MA | mm |
|  | TEG CK LY60 | % reduction in curve area between MA and end of observation interval |
|  | TEG CK R - CKH R | min |

MEA-multiple electrode aggregometry; AUC-area under curve; CT-clot time; α angle- slope of initial clot formation; MCF- maximum clot firmness; ML- maximum clot lysis expressed as percentage of MCF; V_max_ - maximum rate of increase in clot firmness; tV_max_- time to reach maximum rate of increase in clot firmness; CK- TEG citrated kaolin test; CKH- TEG citrated kaolin with heparinase test; R- clot response time; MA- clot maximum amplitude; LY 60- clot lysis at 60 minutes expressed as a percentage reduction in MA.
