# Supplementary material for: Near‐patient coagulation testing to predict bleeding after cardiac surgery: a cohort study
Source: Res Pract Thromb Haemost. 2017 Jul 25;1(2):242–51. doi: 10.1002/rth2.12024 (PMC5992888; doi:10.1002/rth2.12024)
Supplement: Supplementary file 5 [file RTH2-1-242-s005.docx]

**Table S4: Sensitivity analyses.**

| **Sensitivity analyses** | **Rationale** | **Details** |
| --- | --- | --- |
| Sensitivity analyses 1 (SA1) | It is not possible to resolve whether patients who received a pro-haemostatic treatment according to clinician judgement had severe bleeding or no severe bleeding. | Participants who received a pro-haemostatic treatment according to clinician judgement and did not have chest drain bleeding > 600 mL by 6 hours after surgery, or a re-operation for bleeding were excluded from the analysis. |
| Sensitivity analyses 2 (SA2) | Patients who received a pro-haemostatic treatment according to clinician judgement did not have severe bleeding but received this treatment when there was no bleeding or when bleeding was present but not severe. | Participants who received pro-haemostatic treatment according to clinician judgement and did not have chest drain bleeding > 600 mL by 6 hours after surgery, or a re-operation for bleeding, were included in the analysis but were reclassified as not having the primary outcome. |
| Sensitivity analyses 3 (SA3) | Small volume fresh frozen plasma or platelet transfusions may not have been given in response to severe bleeding. | Participants who received pro-haemostatic treatment according to clinician judgement with 1-2 unit fresh frozen plasma transfusion or 1 unit platelet transfusion and did not have chest drain bleeding > 600 mL by 6 hours after surgery or a re-operation for bleeding were included in the analysis but were reclassified as not having the primary outcome. |
