# Supplementary material for: Near‐patient coagulation testing to predict bleeding after cardiac surgery: a cohort study
Source: Res Pract Thromb Haemost. 2017 Jul 25;1(2):242–51. doi: 10.1002/rth2.12024 (PMC5992888; doi:10.1002/rth2.12024)
Supplement: Supplementary file 6 [file RTH2-1-242-s006.docx]

**Table S5: The baseline characteristics of the analysis population and those excluded.**

|  | **Analysis population (n=1833)** | | **Consented, but not in analysis population (n=708)** | | **Standardised mean difference** |
| --- | --- | --- | --- | --- | --- |
| **Age** (median, range) | 68.9 | (18.7, 91.9) | 69.1 | (18.8, 88.2) | 0.09^†^ |
| **Sex;** n (%) male | 1389/1833 | 75.8% | 516/708 | 72.9% | 0.07 |
| **Diabetes;** n (%) | 380/1833 | 20.7% | 119/659 | 18.1% | 0.07 |
| **Procedure and anti-platelet medication;** n (%) |  |  |  |  |  |
| CABG: ASP  CABG: no APT | 737/1833 | 40.2% | 170/536 | 31.7% | 0.18 |
|  | 61/1833 | 3.3% | 18/536 | 3.4% | 0.00 |
| CABG + valve: no APT | 66/1833 | 3.6% | 20/536 | 3.7% | 0.01 |
| Valve: no APT | 301/1833 | 16.4% | 99/536 | 18.5% | 0.05 |
| CABG + valve: ASP | 127/1833 | 6.9% | 49/536 | 9.1% | 0.08 |
| Valve: ASP | 133/1833 | 7.3% | 55/536 | 10.3% | 0.11 |
| CABG: DAPT (0-2 days) | 98/1833 | 5.3% | 30/536 | 5.6% | 0.01 |
| CABG: DAPT (3-5 days) | 114/1833 | 6.2% | 22/536 | 4.1% | 0.09 |
| CABG: DAPT (6-7 days) | 88/1833 | 4.8% | 27/536 | 5.0% | 0.01 |
| CABG + valve: DAPT (≤7days) | 14/1833 | 0.8% | 8/536 | 1.5% | 0.08 |
| Valve: DAPT (≤7days) | 5/1833 | 0.3% | 2/536 | 0.4% | 0.02 |
| Other high risk procedure | 89/1833 | 4.9% | 36/536 | 6.7% | 0.08 |
| **Priority;** n (%) |  |  |  |  |  |
| elective | 1204/1833 | 65.7% | 454/708 | 64.1% | 0.03 |
| urgent | 629/1833 | 34.3% | 254/708 | 35.9% | 0.03 |
| **Pre-operative estimated glomerular filtration rate** (median, range) | 73.7 | (8.0, 214.6) | 73.8 | (9.4, 232.4) | 0.04^†^ |
| **Pre-operative haematocrit** (mean, SD) | 35.94 | 4.4 | 36.11 | 4.4 | 0.04 |
| **Pre-operative platelet count** (median, I range QR) | 205.0 | (43.0, 561.0) | 201.0 | (50.0, 727.0) | 0.04^†^ |
| **Body mass index** (median, range) | 27.4 | (16.2, 64.5) | 27.5 | (17.0, 51.5) | 0.03^†^ |

^†^Standardised mean differences are calculated on means for these characteristics. IQR-interquartile range; CABG-coronary artery bypass grafting; Valve-valve replacement; CABG + valve- combined CABG and valve replacement; ASP- pre-operative aspirin; no APT- no pre-operative anti-platelet medication; DAPT-aspirin + P2Y_12_ blocker shown with duration of omission of P2Y_12_ blocker before surgery; eGFP- estimated glomerular filtration rate; SD standard deviation.
