# Supplementary material for: Near‐patient coagulation testing to predict bleeding after cardiac surgery: a cohort study
Source: Res Pract Thromb Haemost. 2017 Jul 25;1(2):242–51. doi: 10.1002/rth2.12024 (PMC5992888; doi:10.1002/rth2.12024)
Supplement: Supplementary file 7 [file RTH2-1-242-s007.docx]

**Table S6: Near-patient test results**

| **Test**  **(reference interval)** |  | **All analysis population** | | | | **Below reference interval (%)** | | | **Above reference interval (%)** |
| --- | --- | --- | --- | --- | --- | --- | --- | --- | --- |
| **Multiple electrode platelet function analyser** | |  | | | | |  |  |  |
| Pre-op ADP-test AUC; U | (54.0 - 137.6) | 73.8 (27.6) | | | | 450 (24.5%) | | | 23 (1.3%) |
| Pre-op ASPI-test AUC; U | (68.3 - 147.5) | 25.9 (0.8, 157.3) | | | | 1394 (76.1%) | | | 8 (0.4%) |
| Pre-op TRAP-test AUC; U | (92.1 - 165.0) | 118.7 (2.4, 218.5) | | | | 320 (17.5%) | | | 70 (3.8%) |
| Pre-op ADR AUC; U | (35.9 – 78.2) | 25.0 (13.3) | | | | 1547 (84.4%) | | | 10 (0.5%) |
| Post-op ADP-test AUC; U | (54.0 - 137.6) | 49.8 (2.5, 174.6) | | | | 1010 (55.1%) | | | 27 (1.5%) |
| Post-op ASPI-test AUC; U | (68.3 - 147.5) | 19.7 (0.6, 184.9) | | | | 1689 (92.1%) | | | 3 (0.2%) |
| Post-op TRAP-test AUC; U | (92.1 - 165.0) | 115.9 (13.1, 277.7) | | | | 515 (28.1%) | | | 197 (10.7%) |
| Post-op ADR AUC; U | (35.9 – 78.2) | 24.6 (14.4) | | | | 1471 (80.3%) | | | 5 (0.3%) |
| **ROTEM delta thromboelastometer** | | |  |  |  | | | |  |
| INTEM CT; s | (112.0 - 240.8) | 166.0 (104.0, 401.0) | | | | 5 (0.3%) | | | 21 (1.1%) |
| INTEM α angle; ° | (68.3 – 82.7) | 74.0 (44.0, 83.0) | | | | 281 (15.3%) | | | 9 (0.5%) |
| INTEM MCF; mm | (49.2 - 71.7) | 62.0 (36.0, 79.0) | | | | 65 (3.5%) | | | 86 (4.7%) |
| INTEM ML; % | (0-13.1) | 4.0 (0.0, 20.0) | | | | 0 (0%) | | | 54 (2.9%) |
| INTEM V_max_; mm/s | (5.5 - 28.2) | 16.0 (4.0, 40.0) | | | | 2 (0.1%) | | | 57 (3.1%) |
| INTEM tV_max_; s | (111.2 - 282.2) | 202.3 (36.7) | | | | 2 (0.1%) | | | 45 (2.5%) |
| EXTEM CT; s | (37.9 - 73.2) | 56.0 (14.0, 145.0) | | | | 36 (2.0%) | | | 141 (7.7%) |
| EXTEM α angle; ° | (59.1 - 81.9) | 74.0 (48.0, 84.0) | | | | 28 (1.5%) | | | 38 (2.1%) |
| EXTEM MCF; mm | (48.2 - 77.8) | 63.0 (30.0, 82.0) | | | | 27 (1.5%) | | | 14 (0.8%) |
| EXTEM ML; % | (0 - 15.3) | 4.0 (0.0, 31.0) | | | | 0 (0%) | | | 37 (2.0%) |
| EXTEM V_max_; mm/s | (5.9 - 31.9) | 16.0 (5.0, 58.0) | | | | 1 (0.1%) | | | 27 (1.5%) |
| EXTEM tV_max_; s | (46.1 - 162.9) | 102.0 (20.0, 292.0) | | | | 13 (0.7%) | | | 36 (2.0%) |
| FIBTEM MCF; mm | (8.1 - 26.1) | 13.0 (3.0, 42.0) | | | | 222 (12.1%) | | | 48 (2.6%) |
| EXTEM MCF - FIBTEM MCF; mm | (28.4 – 61.2) | 49.0 (14.0, 62.0) | | | | 1 (0.1%) | | | 1 (0.1%) |
| INTEM CT - HEPTEM CT; s | (-68.2 – 81.2) | 0.0 (-92.0, 158.0) | | | | 7 (0.4%) | | | 9 (0.5%) |
| **TEG^®^ 5000 thromboelastograph** |  |  | | | |  | | |  |
| CK R; min | (3.8 - 9.1) | 5.9 (2.5, 18.4) | | | | 40 (2.2%) | | | 74 (4.0%) |
| CK α angle; ° | (47.1 - 76.1) | 63.4 (25.1, 78.7) | | | | 61 (3.3%) | | | 8 (0.4%) |
| CK MA; mm | (51.7 - 72.7) | 58.9 (6.5) | | | | 228 (12.4%) | | | 25 (1.4%) |
| CK LY 60; % | (-3.1 - 6.8) | -0.1 (-11.6, 12.9) | | | | 114 (6.2%) | | | 27 (1.5%) |
| CK R - CKH R; min | (-1.1 - 3.2) | 0.1 (-8.0, 13.2) | | | | 12 (0.7%) | | | 13 (0.7%) |

Data are expressed as median (range) except for Pre-op ADP-test and ADR, Post-op ADR, INT tV_max_ and CK MA which are expressed as mean (standard deviation). AUC- area under curve; CT-clot time; α angle initial slope of clot formation; MCF- maximum clot firmness; ML- maximum clot lysis expressed as the % change in MCF at 60 minutes; V_max_- maximum rate of increase in clot firmness; tV_max_- time to V_max_; CK- TEG with citrated kaolin; CKH- TEG with citrated kaolin and heparinase; R- clot response time; MA- clot maximum amplitude; LY 60 clot lysis expressed as % change in area of the thromboelastogtraphy curve between MA 60 minutes. Test results were compared to a 95% reference interval obtained from 42 healthy volunteers not receiving anti-platelet drugs (median age 48 years, 68% male), to determine the proportions above and below reference interval.
