# Supplementary material for: Near‐patient coagulation testing to predict bleeding after cardiac surgery: a cohort study
Source: Res Pract Thromb Haemost. 2017 Jul 25;1(2):242–51. doi: 10.1002/rth2.12024 (PMC5992888; doi:10.1002/rth2.12024)
Supplement: Supplementary file 8 [file RTH2-1-242-s008.docx]

**Table S7: Performance of predictive models for the primary outcome**

|  | **Baseline-only** | **Baseline-plus-test** | **Test-only** |
| --- | --- | --- | --- |
| **C-statistic** | 0.72 (0.69 to 0.75) | 0.75 (0.72 to 0.77) | 0.71 (0.69 to 0.74) |
| **Percentage correctly classified** | 76.8 | 77.7 | 76.1 |
| **Bootstrapped estimates** | 0.72 (0.69 to 0.75) | 0.75 (0.72 to 0.77) | 0.71 (0.68 to 0.74) |
| **Internal cross-validation** | 0.72 (0.69 to 0.75) | 0.74 (0.72 to 0.77) | 0.70 (0.68 to 0.73) |
| **Hosmer-Lemeshow test** | p=0.59 | p=0.15 | p=0.67 |
