# Supplementary material for: Near‐patient coagulation testing to predict bleeding after cardiac surgery: a cohort study
Source: Res Pract Thromb Haemost. 2017 Jul 25;1(2):242–51. doi: 10.1002/rth2.12024 (PMC5992888; doi:10.1002/rth2.12024)
Supplement: Supplementary file 9 [file RTH2-1-242-s009.docx]

**Table S8: C-statistics of the *baseline + tes*t models for CCB and sensitivity analyses.**

| **Model** | **Original** | **SA 1** | **SA 2** | **SA 3** |
| --- | --- | --- | --- | --- |
| Baseline characteristics | 0.72 (0.69 to 0.75) | 0.72 (0.69 to 0.75) | 0.70 (0.67 to 0.74) | 0.71 (0.68 to 0.74) |
| + pre-op MEA | 0.72 (0.70 to 0.75) | 0.73 (0.69 to 0.76) | 0.71 (0.67 to 0.74) | 0.72 (0.69 to 0.75) |
| + post-op MEA | 0.74 (0.71 to 0.76) | 0.74 (0.70 to 0.77) | 0.72 (0.69 to 0.75) | 0.73 (0.70 to 0.76) |
| + post-op ROTEM | 0.73 (0.71 to 0.76) | 0.74 (0.71 to 0.77) | 0.72 (0.69 to 0.75) | 0.73 (0.70 to 0.76) |
| + post-op TEG | 0.74 (0.71 to 0.76) | 0.74 (0.71 to 0.77) | 0.72 (0.69 to 0.75) | 0.73 (0.70 to 0.76) |
| + post-op ROTEM +post-op MEA | 0.74 (0.72 to 0.77) | 0.73 (0.69 to 0.76) | 0.69 (0.66 to 0.72) | 0.74 (0.71 to 0.77) |
| **+ post-op TEG +post-op MEA** | **0.75 (0.72 to 0.77)** | **0.72 (0.69 to 0.76)** | **0.70 (0.66 to 0.73)** | **0.74 (0.71 to 0.77)** |

The best predictive model is indicated in bold. The figures in the brackets are the 95% confidence intervals of the c-statistics.

**Sensitivity analysis (SA) 1**- Participants classified as CCB because of a pro-haemostatic treatment according to clinician judgement (n=181) are excluded from analysis: rate of CCB = 268/1652 (16.2%).

**Sensitivity analysis (SA) 2**- Participants classified as CCB because of a pro-haemostatic treatment according to clinician judgement only are re-classified as no CCB: rate of CCB = 268/1833 (14.6%).

**Sensitivity analysis (SA) 3**- Participants classified as CCB only because they received 1-2 units of fresh frozen plasma or 1 unit of platelets are re re-classified as no CCB: rate of CCB = 348/1832 (19.0%).
