# Supplementary material for: Near‐patient coagulation testing to predict bleeding after cardiac surgery: a cohort study
Source: Res Pract Thromb Haemost. 2017 Jul 25;1(2):242–51. doi: 10.1002/rth2.12024 (PMC5992888; doi:10.1002/rth2.12024)
Supplement: Supplementary file 10 [file RTH2-1-242-s010.docx]

**Table S9: Near-patient tests that contributed to the best *baseline-plus-test* predictive models**

| **Test and parameter** | | **Units** | **Adjusted OR (95% CI)** | **P value** |
| --- | --- | --- | --- | --- |
| Post-op TEG | CK MA | mm | 0.96 (0.94, 0.98) | <0.001 |
| Post-op MEA | Log_e_ (ADP AUC÷100) | AU | 0.50 (0.37, 0.67) | <0.001 |
| Post-op MEA | ADREN AUC÷10 | AU | 1.19 (1.07, 1.32) | 0.001 |

Results are expressed as odds ratios (OR) with 95% confidence intervals (95% CI) compared with baseline characteristics model (OR=1.0). CK- TEG citrated kaolin test; MA- clot maximum amplitude; ADP AUC- area under curve with MEA ADP test; ADREN AUC- area under curve with MEA and adrenaline reagent; AU-aggregation units.
