# Supplementary material for: Near‐patient coagulation testing to predict bleeding after cardiac surgery: a cohort study
Source: Res Pract Thromb Haemost. 2017 Jul 25;1(2):242–51. doi: 10.1002/rth2.12024 (PMC5992888; doi:10.1002/rth2.12024)
Supplement: Supplementary file 11 [file RTH2-1-242-s011.docx]

**Tables S10: Near-patient test results and secondary outcomes.** AUC- area under curve; SD- standard deviation; IQR- interquartile range; IN- ROTEM INTEM test; EXT- ROTEM EXTEM test; FIB- ROTEM FIBTEM test; HEP- ROTEM HEPTEM test ; CT-clot time; MCF- maximum clot firmness; ML- maximum clot lysis expressed as percentage of MCF; V_max_ - maximum rate of increase in clot firmness; tV_max_- time to reach maximum rate of increase in clot firmness; CK- TEG citrated kaolin test; CKH- TEG citrated kaolin with heparinase test; R- clot response time; MA clot maximum amplitude; LY 60 clot lysis at 60 minutes expressed as a percentage reduction in MA.

**Secondary outcome 1: Red cell transfusion (RBC); 0 vs ≥1 unit (excluding those with surgical cause)**

|  | **RBC intra-op or post-op 0 (n=1137)** | | **RBC intra-op or post-op ≥1 (n=660)** | | **Total (n=1797)** | |
| --- | --- | --- | --- | --- | --- | --- |
| Pre-op ADP-test AUC; mean (SD); U | 740.0 | 264.0 | 742.9 | 292.2 | 741.1 | 274.6 |
| Pre-op ASPI-test AUC; median (range); U | 270.0 | (15.0, 1546.0) | 251.5 | (8.0, 1573.0) | 261.0 | (8.0, 1573.0) |
| Pre-op TRAP-test AUC; median (range); U | 1199.0 | (51.0, 2139.0) | 1176.0 | (215.0, 2185.0) | 1187.0 | (51.0, 2185.0) |
| Pre-op ADR AUC; mean (SD); U | 250.3 | 130.3 | 252.3 | 138.9 | 251.0 | 133.5 |
|  | **RBC post-op 0 (n=1214)** | | **RBC post-op RBC ≥1 (n=581)** | | **Total (n=1795)** | |
| Pre-op ADP-test AUC; mean (SD); U | 271.0 | (15.0, 1573.0) | 246.0 | (8.0, 1567.0) | 261.0 | (8.0, 1573.0) |
| Pre-op ASPI-test AUC; median (range); U | 1190.0 | (51.0, 2139.0) | 1174.0 | (215.0, 2185.0) | 1187.0 | (51.0, 2185.0) |
| Pre-op TRAP-test AUC; mean (IQR); U | 248.5 | 129.9 | 255.5 | 139.7 | 250.8 | 133.2 |
| Pre-op ADR AUC; median (SD); U | 542.5 | (32.0, 1746.0) | 424.0 | (25.0, 1607.0) | 501.0 | (25.0, 1746.0) |
| Post-op ADP-test AUC; median (range); U | 208.0 | (8.0, 1609.0) | 176.0 | (12.0, 1849.0) | 197.0 | (8.0, 1849.0) |
| Post-op ASPI-test AUC; median (range); U | 1210.0 | (131.0, 2777.0) | 1055.0 | (213.0, 2395.0) | 1159.0 | (131.0, 2777.0) |
| Post-op TRAP-test AUC; median (range); U | 251.0 | 139.6 | 236.9 | 150.2 | 246.4 | 143.2 |
| Post-op ADR AUC; mean (SD); U | 271.0 | (15.0, 1573.0) | 246.0 | (8.0, 1567.0) | 261.0 | (8.0, 1573.0) |
| intem CT; median (range); s | 166.0 | (106.0, 401.0) | 166.0 | (104.0, 327.0) | 166.0 | (104.0, 401.0) |
| intem α angle; median (range); ° | 75.0 | (51.0, 83.0) | 74.0 | (44.0, 83.0) | 74.0 | (44.0, 83.0) |
| intem MCF; median (range); mm | 62.0 | (40.0, 78.0) | 61.0 | (36.0, 79.0) | 62.0 | (36.0, 79.0) |
| intem ML; median (range); % | 5.0 | (0.0, 20.0) | 4.0 | (0.0, 20.0) | 4.0 | (0.0, 20.0) |
| intem V_max_; median (range); mm/s | 16.0 | (6.0, 39.0) | 15.0 | (4.0, 40.0) | 16.0 | (4.0, 40.0) |
| intem tV_max_; mean (SD); s | 201.9 | 35.3 | 202.6 | 39.3 | 202.1 | 36.6 |
| extem CT; median (range); s | 56.0 | (14.0, 120.0) | 57.0 | (20.0, 145.0) | 56.0 | (14.0, 145.0) |
| extem α angle; median (range); ° | 74.0 | (50.0, 84.0) | 73.0 | (48.0, 84.0) | 74.0 | (48.0, 84.0) |
| extem MCF; median (range); mm | 63.0 | (30.0, 82.0) | 63.0 | (37.0, 79.0) | 63.0 | (30.0, 82.0) |
| extem ML; median (range); % | 5.0 | (0.0, 31.0) | 4.0 | (0.0, 26.0) | 4.0 | (0.0, 31.0) |
| extem V_max_; median (range); mm/s | 16.0 | (6.0, 52.0) | 15.0 | (5.0, 58.0) | 16.0 | (5.0, 58.0) |
| extem tV_max_; median (range); s | 101.0 | (20.0, 254.0) | 103.0 | (40.0, 292.0) | 103.0 | (20.0, 292.0) |
| fibtem MCF; median (range); mm | 13.0 | (3.0, 40.0) | 13.0 | (3.0, 42.0) | 13.0 | (3.0, 42.0) |
| extem MCF-fibtem MCF; median (range); mm | 49.0 | (14.0, 62.0) | 49.0 | (32.0, 61.0) | 49.0 | (14.0, 62.0) |
| intem CT - heptem CT; median (range); s | 0.0 | (-92.0, 158.0) | 0.0 | (-89.0, 91.0) | 0.0 | (-92.0, 158.0) |
| CK R; median (range); min | 6.2 | (2.7, 15.7) | 5.7 | (2.5, 18.4) | 5.9 | (2.5, 18.4) |
| CK α angle ; median (range); ° | 62.8 | (31.3, 76.7) | 65.1 | (25.1, 78.7) | 63.4 | (25.1, 78.7) |
| CK MA; mean (SD); mm | 59.1 | 5.9 | 58.5 | 7.4 | 58.9 | 6.5 |
| CK LY 60; median (range); % | -0.2 | (-11.6, 12.9) | -0.1 | (-11.1, 7.7) | -0.1 | (-11.6, 12.9) |
| CK R - CKH R; median (range); min | 0.2 | (-2.4, 8.3) | 0.1 | (-8.0, 13.2) | 0.1 | (-8.0, 13.2) |

**Secondary outcome 2: Red cell transfusion (RBC); ≤4 units vs >4 units (excluding those with surgical cause)**

|  | **RBC ≤4 intra-op or post-op (n=1700)** | | **RBC >4 intra-op or post-op (n=96)** | | **Total (n=1796)** | |
| --- | --- | --- | --- | --- | --- | --- |
| Pre-op ADP-test AUC; mean (SD); U | 743.5 | 272.7 | 697.2 | 305.9 | 741.1 | 274.7 |
| Pre-op ASPI-test AUC; median (range); U | 261.0 | (14.0, 1573.0) | 252.5 | (8.0, 1508.0) | 260.5 | (8.0, 1573.0) |
| Pre-op TRAP-test AUC; median (range); U | 1188.0 | (51.0, 2139.0) | 1135.0 | (215.0, 2185.0) | 1187.0 | (51.0, 2185.0) |
| Pre-op ADR AUC; mean (SD); U | 250.4 | 133.5 | 261.9 | 134.3 | 251.0 | 133.5 |
|  | **RBC ≤4 intra-op or post-op (n=1743)** | | **RBC >4 intra-op or post-op (n=59)** | | **Total (n=1802)** | |
| Pre-op ADP-test AUC; mean (SD); U | 741.9 | 275.1 | 715.0 | 251.5 | 741.0 | 274.3 |
| Pre-op ASPI-test AUC; median (range); U | 260.0 | (8.0, 1573.0) | 299.0 | (33.0, 1318.0) | 260.5 | (8.0, 1573.0) |
| Pre-op TRAP-test AUC; mean (IQR); U | 1187.0 | (51.0, 2185.0) | 1177.0 | (527.0, 1728.0) | 1187.0 | (51.0, 2185.0) |
| Pre-op ADR AUC; median (SD); U | 250.0 | 132.9 | 271.8 | 147.7 | 250.7 | 133.4 |
| Post-op ADP-test AUC; median (range); U | 502.0 | (25.0, 1746.0) | 441.0 | (192.0, 1297.0) | 500.5 | (25.0, 1746.0) |
| Post-op ASPI-test AUC; median (range); U | 197.0 | (8.0, 1849.0) | 200.0 | (6.0, 1314.0) | 197.0 | (6.0, 1849.0) |
| Post-op TRAP-test AUC; median (range); U | 1163.0 | (131.0, 2777.0) | 1027.0 | (311.0, 1904.0) | 1160.0 | (131.0, 2777.0) |
| Post-op ADR AUC; mean (SD); U | 245.8 | 141.8 | 266.8 | 189.3 | 246.5 | 143.5 |
| intem CT; median (range); s | 166.0 | (104.0, 401.0) | 164.0 | (128.0, 269.0) | 166.0 | (104.0, 401.0) |
| intem α angle; median (range); ° | 74.0 | (44.0, 83.0) | 74.0 | (48.0, 83.0) | 74.0 | (44.0, 83.0) |
| intem MCF; median (range); mm | 62.0 | (36.0, 79.0) | 62.0 | (41.0, 77.0) | 62.0 | (36.0, 79.0) |
| intem ML; median (range); % | 5.0 | (0.0, 20.0) | 3.0 | (0.0, 12.0) | 4.0 | (0.0, 20.0) |
| intem V_max_; median (range); mm/s | 16.0 | (4.0, 40.0) | 16.0 | (6.0, 36.0) | 16.0 | (4.0, 40.0) |
| intem tV_max_; mean (SD); s | 202.2 | 36.6 | 200.7 | 36.5 | 202.1 | 36.6 |
| extem CT; median (range); s | 56.0 | (14.0, 145.0) | 55.0 | (34.0, 81.0) | 56.0 | (14.0, 145.0) |
| extem α angle; median (range); ° | 74.0 | (48.0, 84.0) | 73.0 | (57.0, 84.0) | 74.0 | (48.0, 84.0) |
| extem MCF; median (range); mm | 63.0 | (30.0, 82.0) | 63.0 | (44.0, 79.0) | 63.0 | (30.0, 82.0) |
| extem ML; median (range); % | 5.0 | (0.0, 31.0) | 3.0 | (0.0, 13.0) | 4.0 | (0.0, 31.0) |
| extem V_max_; median (range); mm/s | 16.0 | (5.0, 58.0) | 15.0 | (8.0, 42.0) | 16.0 | (5.0, 58.0) |
| extem tV_max_; median (range); s | 103.0 | (20.0, 254.0) | 95.0 | (44.0, 292.0) | 103.0 | (20.0, 292.0) |
| fibtem MCF; median (range); mm | 13.0 | (3.0, 40.0) | 14.0 | (6.0, 42.0) | 13.0 | (3.0, 42.0) |
| extem MCF-fibtem MCF; median (range); mm | 49.0 | (14.0, 62.0) | 48.0 | (34.0, 56.0) | 49.0 | (14.0, 62.0) |
| intem CT - heptem CT; median (range); s | 0.0 | (-92.0, 158.0) | -2.0 | (-57.0, 91.0) | 0.0 | (-92.0, 158.0) |
| CK R; median (range); min | 5.9 | (2.5, 18.4) | 5.8 | (3.3, 14.1) | 5.9 | (2.5, 18.4) |
| CK α angle ; median (range); ° | 63.4 | (25.1, 78.7) | 65.8 | (34.4, 76.0) | 63.4 | (25.1, 78.7) |
| CK MA; mean (SD); mm | 58.9 | 6.4 | 59.2 | 8.1 | 58.9 | 6.5 |
| CK LY 60; median (range); % | -0.1 | (-11.6, 12.9) | -0.1 | (-6.9, 3.9) | -0.1 | (-11.6, 12.9) |
| CK R - CKH R; median (range); min | 0.1 | (-8.0, 13.2) | 0.1 | (-0.7, 4.8) | 0.1 | (-8.0, 13.2) |

**Secondary outcome 3: Mortality**

|  | **No death (n=1799)** | | **Death (n=34)** | | **Total (n=1833)** | |
| --- | --- | --- | --- | --- | --- | --- |
| Pre-op ADP-test AUC; mean (SD); U | 739.0 | 274.6 | 710.3 | 325.6 | 738.5 | 275.5 |
| Pre-op ASPI-test AUC; median (range); U | 259.0 | (8.0, 1573.0) | 291.0 | (46.0, 1422.0) | 259.0 | (8.0, 1573.0) |
| Pre-op TRAP-test AUC; mean (IQR); U | 1187.0 | (24.0, 2139.0) | 1161.5 | (352.0, 2185.0) | 1187.0 | (24.0, 2185.0) |
| Pre-op ADR AUC; median (SD); U | 249.9 | 133.1 | 264.1 | 137.6 | 250.1 | 133.2 |
| Post-op ADP-test AUC; median (range); U | 501.0 | (25.0, 1746.0) | 342.0 | (129.0, 1039.0) | 498.0 | (25.0, 1746.0) |
| Post-op ASPI-test AUC; median (range); U | 197.0 | (6.0, 1849.0) | 170.0 | (25.0, 1350.0) | 197.0 | (6.0, 1849.0) |
| Post-op TRAP-test AUC; median (range); U | 1161.0 | (131.0, 2777.0) | 963.5 | (277.0, 1794.0) | 1159.0 | (131.0, 2777.0) |
| Post-op ADR AUC; mean (SD); U | 246.8 | 143.4 | 223.2 | 155.8 | 246.4 | 143.6 |
| intem CT; median (range); s | 166.0 | (104.0, 401.0) | 162.0 | (132.0, 216.0) | 166.0 | (104.0, 401.0) |
| intem α angle; median (range); ° | 74.0 | (44.0, 83.0) | 76.0 | (58.0, 82.0) | 74.0 | (44.0, 83.0) |
| intem MCF; median (range); mm | 61.0 | (36.0, 79.0) | 62.5 | (49.0, 74.0) | 62.0 | (36.0, 79.0) |
| intem ML; median (range); % | 5.0 | (0.0, 20.0) | 2.0 | (0.0, 14.0) | 4.0 | (0.0, 20.0) |
| intem V_max_; median (range); mm/s | 16.0 | (4.0, 40.0) | 18.0 | (7.0, 31.0) | 16.0 | (4.0, 40.0) |
| intem tV_max_; mean (SD); s | 202.4 | 36.8 | 196.6 | 31.8 | 202.3 | 36.7 |
| extem CT; median (range); s | 56.0 | (14.0, 145.0) | 57.0 | (24.0, 93.0) | 56.0 | (14.0, 145.0) |
| extem α angle; median (range); ° | 74.0 | (48.0, 84.0) | 74.0 | (57.0, 81.0) | 74.0 | (48.0, 84.0) |
| extem MCF; median (range); mm | 63.0 | (30.0, 82.0) | 63.5 | (51.0, 76.0) | 63.0 | (30.0, 82.0) |
| extem ML; median (range); % | 4.0 | (0.0, 31.0) | 3.0 | (0.0, 14.0) | 4.0 | (0.0, 31.0) |
| extem V_max_; median (range); mm/s | 16.0 | (5.0, 58.0) | 16.0 | (8.0, 27.0) | 16.0 | (5.0, 58.0) |
| extem tV_max_; median (range); s | 103.0 | (20.0, 292.0) | 99.0 | (52.0, 157.0) | 102.0 | (20.0, 292.0) |
| fibtem MCF; median (range); mm | 13.0 | (3.0, 42.0) | 13.5 | (4.0, 24.0) | 13.0 | (3.0, 42.0) |
| extem MCF-fibtem MCF; median (range); mm | 49.0 | (14.0, 62.0) | 49.5 | (41.0, 57.0) | 49.0 | (14.0, 62.0) |
| intem CT - heptem CT; median (range); s | 0.0 | (-92.0, 158.0) | 5.5 | (-49.0, 41.0) | 0.0 | (-92.0, 158.0) |
| CK R; median (range); min | 5.9 | (2.5, 18.4) | 5.8 | (3.3, 9.6) | 5.9 | (2.5, 18.4) |
| CK α angle ; median (range); ° | 63.3 | (25.1, 78.7) | 66.3 | (45.8, 74.0) | 63.4 | (25.1, 78.7) |
| CK MA; mean (SD); mm | 58.9 | 6.4 | 59.2 | 7.8 | 58.9 | 6.5 |
| CK LY 60; median (range); % | -0.1 | (-11.6, 12.9) | -0.1 | (-1.8, 6.1) | -0.1 | (-11.6, 12.9) |
| CK R - CKH R; median (range); min | 0.1 | (-8.0, 13.2) | 0.1 | (-1.3, 2.7) | 0.1 | (-8.0, 13.2) |

**Secondary outcome 4: Myocardial infarction (MI)**

| **Post-op measurements** | **No MI (n=1809)** | | **MI (n=19)** | | **Total (n=1828)** | |
| --- | --- | --- | --- | --- | --- | --- |
| Pre-op ADP-test AUC; mean (SD); U | 738.5 | 275.5 | 746.1 | 315.9 | 738.6 | 275.8 |
| Pre-op ASPI-test AUC; median (range); U | 259.0 | (8.0, 1573.0) | 266.0 | (67.0, 1147.0) | 259.0 | (8.0, 1573.0) |
| Pre-op TRAP-test AUC; mean (IQR); U | 1187.0 | (24.0, 2185.0) | 1166.0 | (598.0, 1783.0) | 1185.5 | (24.0, 2185.0) |
| Pre-op ADR AUC; median (SD); U | 250.4 | 133.2 | 252.9 | 131.2 | 250.4 | 133.2 |
| Post-op ADP-test AUC; median (range); U | 499.0 | (25.0, 1746.0) | 466.0 | (75.0, 1103.0) | 498.0 | (25.0, 1746.0) |
| Post-op ASPI-test AUC; median (range); U | 197.0 | (8.0, 1849.0) | 188.0 | (25.0, 545.0) | 197.0 | (8.0, 1849.0) |
| Post-op TRAP-test AUC; median (range); U | 1157.0 | (131.0, 2777.0) | 1338.0 | (568.0, 1722.0) | 1158.5 | (131.0, 2777.0) |
| Post-op ADR AUC; mean (SD); U | 246.3 | 143.7 | 269.0 | 135.0 | 246.5 | 143.6 |
| intem CT; median (range); s | 166.0 | (104.0, 401.0) | 157.0 | (133.0, 228.0) | 166.0 | (104.0, 401.0) |
| intem α angle; median (range); ° | 74.0 | (44.0, 83.0) | 74.0 | (67.0, 81.0) | 74.0 | (44.0, 83.0) |
| intem MCF; median (range); mm | 62.0 | (36.0, 79.0) | 60.0 | (54.0, 73.0) | 62.0 | (36.0, 79.0) |
| intem ML; median (range); % | 4.0 | (0.0, 20.0) | 5.0 | (1.0, 14.0) | 4.0 | (0.0, 20.0) |
| intem V_max_; median (range); mm/s | 16.0 | (4.0, 40.0) | 16.0 | (11.0, 28.0) | 16.0 | (4.0, 40.0) |
| intem tV_max_; mean (SD); s | 202.3 | 36.8 | 196.5 | 29.4 | 202.2 | 36.7 |
| extem CT; median (range); s | 56.0 | (14.0, 145.0) | 50.0 | (35.0, 85.0) | 56.0 | (14.0, 145.0) |
| extem α angle; median (range); ° | 74.0 | (48.0, 84.0) | 74.0 | (67.0, 81.0) | 74.0 | (48.0, 84.0) |
| extem MCF; median (range); mm | 63.0 | (30.0, 82.0) | 63.0 | (54.0, 73.0) | 63.0 | (30.0, 82.0) |
| extem ML; median (range); % | 4.0 | (0.0, 31.0) | 5.0 | (0.0, 15.0) | 4.0 | (0.0, 31.0) |
| extem V_max_; median (range); mm/s | 16.0 | (5.0, 58.0) | 16.0 | (12.0, 25.0) | 16.0 | (5.0, 58.0) |
| extem tV_max_; median (range); s | 103.0 | (20.0, 292.0) | 100.0 | (55.0, 162.0) | 102.5 | (20.0, 292.0) |
| fibtem MCF; median (range); mm | 13.0 | (3.0, 42.0) | 13.0 | (5.0, 27.0) | 13.0 | (3.0, 42.0) |
| extem MCF-fibtem MCF; median (range); mm | 49.0 | (14.0, 62.0) | 50.0 | (45.0, 55.0) | 49.0 | (14.0, 62.0) |
| intem CT - heptem CT; median (range); s | 0.0 | (-92.0, 158.0) | 0.0 | (-20.0, 41.0) | 0.0 | (-92.0, 158.0) |
| CK R; median (range); min | 5.9 | (2.5, 18.4) | 5.5 | (4.2, 8.2) | 5.9 | (2.5, 18.4) |
| CK α angle ; median (range); ° | 63.4 | (25.1, 78.7) | 65.1 | (49.1, 73.9) | 63.4 | (25.1, 78.7) |
| CK MA; mean (SD); mm | 58.9 | 6.5 | 60.1 | 5.7 | 58.9 | 6.5 |
| CK LY 60; median (range); % | -0.1 | (-11.6, 12.9) | -0.1 | (-3.9, 5.7) | -0.1 | (-11.6, 12.9) |
| CK R - CKH R; median (range); min | 0.1 | (-8.0, 13.2) | 0.1 | (-1.3, 1.8) | 0.1 | (-8.0, 13.2) |

**Secondary outcome 5: Stroke**

| **Post-op measurements** | **No Stoke (n=1809)** | | **Stroke (n=19)** | | **Total (n=1828)** | |
| --- | --- | --- | --- | --- | --- | --- |
| Pre-op ADP-test AUC; mean (SD); U | 736.7 | 276.0 | 913.0 | 203.3 | 738.6 | 275.8 |
| Pre-op ASPI-test AUC; median (range); U | 258.0 | (8.0, 1573.0) | 636.0 | (22.0, 1147.0) | 259.0 | (8.0, 1573.0) |
| Pre-op TRAP-test AUC; mean (IQR); U | 1184.0 | (24.0, 2185.0) | 1319.0 | (850.0, 1728.0) | 1185.5 | (24.0, 2185.0) |
| Pre-op ADR AUC; median (SD); U | 250.3 | 133.3 | 259.3 | 122.2 | 250.4 | 133.2 |
| Post-op ADP-test AUC; median (range); U | 500.0 | (25.0, 1746.0) | 386.0 | (159.0, 996.0) | 498.0 | (25.0, 1746.0) |
| Post-op ASPI-test AUC; median (range); U | 197.0 | (8.0, 1849.0) | 191.0 | (85.0, 727.0) | 197.0 | (8.0, 1849.0) |
| Post-op TRAP-test AUC; median (range); U | 1159.0 | (131.0, 2777.0) | 1027.0 | (466.0, 1781.0) | 1158.5 | (131.0, 2777.0) |
| Post-op ADR AUC; mean (SD); U | 246.7 | 143.7 | 231.5 | 135.0 | 246.5 | 143.6 |
| intem CT; median (range); s | 166.0 | (104.0, 401.0) | 164.0 | (133.0, 185.0) | 166.0 | (104.0, 401.0) |
| intem α angle; median (range); ° | 74.0 | (44.0, 83.0) | 74.0 | (66.0, 81.0) | 74.0 | (44.0, 83.0) |
| intem MCF; median (range); mm | 62.0 | (36.0, 79.0) | 61.0 | (51.0, 71.0) | 62.0 | (36.0, 79.0) |
| intem ML; median (range); % | 4.0 | (0.0, 20.0) | 3.0 | (0.0, 10.0) | 4.0 | (0.0, 20.0) |
| intem V_max_; median (range); mm/s | 16.0 | (4.0, 40.0) | 15.0 | (10.0, 26.0) | 16.0 | (4.0, 40.0) |
| intem tV_max_; mean (SD); s | 202.3 | 36.8 | 196.8 | 24.0 | 202.2 | 36.7 |
| extem CT; median (range); s | 56.0 | (14.0, 145.0) | 53.0 | (34.0, 118.0) | 56.0 | (14.0, 145.0) |
| extem α angle; median (range); ° | 74.0 | (48.0, 84.0) | 73.0 | (63.0, 82.0) | 74.0 | (48.0, 84.0) |
| extem MCF; median (range); mm | 63.0 | (30.0, 82.0) | 65.0 | (53.0, 72.0) | 63.0 | (30.0, 82.0) |
| extem ML; median (range); % | 4.0 | (0.0, 31.0) | 3.0 | (0.0, 9.0) | 4.0 | (0.0, 31.0) |
| extem V_max_; median (range); mm/s | 16.0 | (5.0, 58.0) | 16.0 | (9.0, 30.0) | 16.0 | (5.0, 58.0) |
| extem tV_max_; median (range); s | 103.0 | (20.0, 292.0) | 86.0 | (40.0, 148.0) | 102.5 | (20.0, 292.0) |
| fibtem MCF; median (range); mm | 13.0 | (3.0, 42.0) | 12.0 | (7.0, 29.0) | 13.0 | (3.0, 42.0) |
| extem MCF-fibtem MCF; median (range); mm | 49.0 | (14.0, 62.0) | 49.0 | (43.0, 55.0) | 49.0 | (14.0, 62.0) |
| intem CT - heptem CT; median (range); s | 0.0 | (-92.0, 158.0) | 0.0 | (-30.0, 14.0) | 0.0 | (-92.0, 158.0) |
| CK R; median (range); min | 5.9 | (2.5, 18.4) | 5.4 | (3.5, 9.5) | 5.9 | (2.5, 18.4) |
| CK α angle ; median (range); ° | 63.4 | (25.1, 78.7) | 66.8 | (55.1, 76.3) | 63.4 | (25.1, 78.7) |
| CK MA; mean (SD); mm | 58.9 | 6.5 | 60.1 | 5.8 | 58.9 | 6.5 |
| CK LY 60; median (range); % | -0.1 | (-11.6, 12.9) | -0.1 | (-3.4, 3.9) | -0.1 | (-11.6, 12.9) |
| CK R - CKH R; median (range); min | 0.1 | (-8.0, 13.2) | 0.0 | (-0.6, 0.9) | 0.1 | (-8.0, 13.2) |

**Secondary outcome 6: Acute kidney injury (AKI)**

| **Post-op measurements** | **No AKI (n=1014)** | | **AKI (n=819)** | | **Total (n=1833** | |
| --- | --- | --- | --- | --- | --- | --- |
| Pre-op ADP-test AUC; mean (SD); U | 735.5 | 264.7 | 742.2 | 288.5 | 738.5 | 275.5 |
| Pre-op ASPI-test AUC; median (range); U | 267.5 | (8.0, 1573.0) | 253.0 | (19.0, 1553.0) | 259.0 | (8.0, 1573.0) |
| Pre-op TRAP-test AUC; mean (IQR); U | 1194.0 | (51.0, 2139.0) | 1174.0 | (24.0, 2185.0) | 1187.0 | (24.0, 2185.0) |
| Pre-op ADR AUC; median (SD); U | 246.3 | 130.5 | 254.9 | 136.3 | 250.1 | 133.2 |
| Post-op ADP-test AUC; median (range); U | 503.0 | (32.0, 1746.0) | 491.0 | (25.0, 1669.0) | 498.0 | (25.0, 1746.0) |
| Post-op ASPI-test AUC; median (range); U | 199.0 | (8.0, 1849.0) | 193.0 | (6.0, 1457.0) | 197.0 | (6.0, 1849.0) |
| Post-op TRAP-test AUC; median (range); U | 1178.5 | (131.0, 2395.0) | 1138.0 | (213.0, 2777.0) | 1159.0 | (131.0, 2777.0) |
| Post-op ADR AUC; mean (SD); U | 244.5 | 146.5 | 248.8 | 140.0 | 246.4 | 143.6 |
| intem CT; median (range); s | 166.0 | (104.0, 327.0) | 166.0 | (111.0, 401.0) | 166.0 | (104.0, 401.0) |
| intem α angle; median (range); ° | 74.0 | (48.0, 83.0) | 74.0 | (44.0, 83.0) | 74.0 | (44.0, 83.0) |
| intem MCF; median (range); mm | 61.0 | (37.0, 79.0) | 62.0 | (36.0, 79.0) | 62.0 | (36.0, 79.0) |
| intem ML; median (range); % | 5.0 | (0.0, 20.0) | 4.0 | (0.0, 20.0) | 4.0 | (0.0, 20.0) |
| intem V_max_; median (range); mm/s | 16.0 | (6.0, 39.0) | 16.0 | (4.0, 40.0) | 16.0 | (4.0, 40.0) |
| intem tV_max_; mean (SD); s | 201.4 | 35.8 | 203.3 | 37.9 | 202.3 | 36.7 |
| extem CT; median (range); s | 56.0 | (16.0, 145.0) | 56.0 | (14.0, 140.0) | 56.0 | (14.0, 145.0) |
| extem α angle; median (range); ° | 73.0 | (50.0, 84.0) | 74.0 | (48.0, 84.0) | 74.0 | (48.0, 84.0) |
| extem MCF; median (range); mm | 63.0 | (30.0, 80.0) | 64.0 | (38.0, 82.0) | 63.0 | (30.0, 82.0) |
| extem ML; median (range); % | 5.0 | (0.0, 21.0) | 4.0 | (0.0, 31.0) | 4.0 | (0.0, 31.0) |
| extem V_max_; median (range); mm/s | 16.0 | (6.0, 58.0) | 16.0 | (5.0, 44.0) | 16.0 | (5.0, 58.0) |
| extem tV_max_; median (range); s | 103.0 | (20.0, 254.0) | 101.0 | (40.0, 292.0) | 102.0 | (20.0, 292.0) |
| fibtem MCF; median (range); mm | 13.0 | (3.0, 38.0) | 13.0 | (3.0, 42.0) | 13.0 | (3.0, 42.0) |
| extem MCF-fibtem MCF; median (range); mm | 49.0 | (14.0, 59.0) | 49.0 | (31.0, 62.0) | 49.0 | (14.0, 62.0) |
| intem CT - heptem CT; median (range); s | 0.0 | (-92.0, 158.0) | -1.0 | (-73.0, 99.0) | 0.0 | (-92.0, 158.0) |
| CK R; median (range); min | 6.0 | (2.5, 14.7) | 5.9 | (2.9, 18.4) | 5.9 | (2.5, 18.4) |
| CK α angle ; median (range); ° | 62.6 | (31.3, 77.1) | 64.4 | (25.1, 78.7) | 63.4 | (25.1, 78.7) |
| CK MA; mean (SD); mm | 58.4 | 6.1 | 59.5 | 6.8 | 58.9 | 6.5 |
| CK LY 60; median (range); % | -0.2 | (-11.6, 12.9) | -0.1 | (-11.1, 12.8) | -0.1 | (-11.6, 12.9) |
| CK R - CKH R; median (range); min | 0.1 | (-8.0, 7.1) | 0.1 | (-2.4, 13.2) | 0.1 | (-8.0, 13.2) |

**Secondary outcome 7: Sepsis**

| **Post-op measurements** | **No sepsis (n=1722)** | | **Sepsis (n=104)** | | **Total (n=1826)** | |
| --- | --- | --- | --- | --- | --- | --- |
| Pre-op ADP-test AUC; mean (SD); U | 739.0 | 275.6 | 735.0 | 280.6 | 738.8 | 275.8 |
| Pre-op ASPI-test AUC; median (range); U | 258.0 | (8.0, 1573.0) | 279.5 | (26.0, 1104.0) | 259.0 | (8.0, 1573.0) |
| Pre-op TRAP-test AUC; mean (IQR); U | 1188.0 | (24.0, 2185.0) | 1168.0 | (215.0, 1844.0) | 1187.0 | (24.0, 2185.0) |
| Pre-op ADR AUC; median (SD); U | 249.6 | 132.8 | 263.4 | 141.4 | 250.4 | 133.3 |
| Post-op ADP-test AUC; median (range); U | 500.0 | (25.0, 1746.0) | 477.0 | (169.0, 1561.0) | 498.5 | (25.0, 1746.0) |
| Post-op ASPI-test AUC; median (range); U | 197.0 | (8.0, 1849.0) | 195.0 | (28.0, 1350.0) | 197.0 | (8.0, 1849.0) |
| Post-op TRAP-test AUC; median (range); U | 1164.0 | (131.0, 2777.0) | 1068.0 | (306.0, 2340.0) | 1158.5 | (131.0, 2777.0) |
| Post-op ADR AUC; mean (SD); U | 246.5 | 144.6 | 246.7 | 128.3 | 246.5 | 143.6 |
| intem CT; median (range); s | 166.0 | (104.0, 401.0) | 167.0 | (131.0, 267.0) | 166.0 | (104.0, 401.0) |
| intem α angle; median (range); ° | 74.0 | (48.0, 83.0) | 75.0 | (44.0, 83.0) | 74.0 | (44.0, 83.0) |
| intem MCF; median (range); mm | 61.0 | (37.0, 79.0) | 63.5 | (36.0, 78.0) | 62.0 | (36.0, 79.0) |
| intem ML; median (range); % | 5.0 | (0.0, 20.0) | 3.0 | (0.0, 14.0) | 4.0 | (0.0, 20.0) |
| intem V_max_; median (range); mm/s | 16.0 | (5.0, 40.0) | 18.0 | (4.0, 38.0) | 16.0 | (4.0, 40.0) |
| intem tV_max_; mean (SD); s | 202.1 | 36.8 | 204.3 | 36.0 | 202.2 | 36.7 |
| extem CT; median (range); s | 56.0 | (14.0, 145.0) | 56.0 | (24.0, 110.0) | 56.0 | (14.0, 145.0) |
| extem α angle; median (range); ° | 74.0 | (50.0, 84.0) | 75.0 | (48.0, 84.0) | 74.0 | (48.0, 84.0) |
| extem MCF; median (range); mm | 63.0 | (30.0, 81.0) | 65.0 | (38.0, 82.0) | 63.0 | (30.0, 82.0) |
| extem ML; median (range); % | 4.5 | (0.0, 26.0) | 3.5 | (0.0, 31.0) | 4.0 | (0.0, 31.0) |
| extem V_max_; median (range); mm/s | 16.0 | (6.0, 58.0) | 17.0 | (5.0, 42.0) | 16.0 | (5.0, 58.0) |
| extem tV_max_; median (range); s | 101.0 | (20.0, 292.0) | 103.0 | (50.0, 196.0) | 102.0 | (20.0, 292.0) |
| fibtem MCF; median (range); mm | 13.0 | (3.0, 42.0) | 15.0 | (3.0, 36.0) | 13.0 | (3.0, 42.0) |
| extem MCF-fibtem MCF; median (range); mm | 49.0 | (14.0, 59.0) | 49.0 | (35.0, 62.0) | 49.0 | (14.0, 62.0) |
| intem CT - heptem CT; median (range); s | 0.0 | (-92.0, 158.0) | 0.0 | (-57.0, 69.0) | 0.0 | (-92.0, 158.0) |
| CK R; median (range); min | 6.0 | (2.5, 18.4) | 5.8 | (2.8, 11.4) | 5.9 | (2.5, 18.4) |
| CK α angle ; median (range); ° | 63.2 | (25.1, 78.7) | 65.8 | (38.9, 76.7) | 63.4 | (25.1, 78.7) |
| CK MA; mean (SD); mm | 58.8 | 6.4 | 60.4 | 7.3 | 58.9 | 6.5 |
| CK LY 60; median (range); % | -0.1 | (-11.6, 12.9) | -0.1 | (-4.4, 7.0) | -0.1 | (-11.6, 12.9) |
| CK R - CKH R; median (range); min | 0.1 | (-8.0, 13.2) | 0.1 | (-2.3, 1.5) | 0.1 | (-8.0, 13.2) |
