# Supplementary material for: Near‐patient coagulation testing to predict bleeding after cardiac surgery: a cohort study
Source: Res Pract Thromb Haemost. 2017 Jul 25;1(2):242–51. doi: 10.1002/rth2.12024 (PMC5992888; doi:10.1002/rth2.12024)
Supplement: Supplementary file 12 [file RTH2-1-242-s012.docx]

**SUPPLEMENTARY INFORMATION**

| **Table S1** | Secondary endpoint definitions | |
| --- | --- | --- |
| **Table S2** | Baseline characteristic predictors | |
| **Table S3** | Near patient test predictors | |
| **Table S4** | Sensitivity analyses | |
| **Table S5** | Baseline characteristics of the analysis population and those excluded | |
| **Table S6** | Near patient test results | |
| **Table S7** | Performance of predictive models for the primary outcome | |
| **Table S8** | C-statistics of *baseline-plus-test* models and sensitivity analyses | |
| **Table S9** | Near-patient tests that contributed to the best *baseline-plus-test* predictive models | |
| **Table S10** | Near-patient test results and secondary outcomes | |
| **Figure S1** | Proportions of the analysis population with components of CCB | |
| **Figure S2** | Distributions of near patient test results in patients with CCB and patients without CCB | |
| **Document S1** | Study protocol |  |
